# Supplementary material for: Investigation of the Antihypertrophic and Antifibrotic Effects of Losartan in a Rat Model of Radiation-Induced Heart Disease
Source: Int J Mol Sci. 2021 Nov 30;22(23):12963. doi: 10.3390/ijms222312963 (PMC8657420; doi:10.3390/ijms222312963)
Supplement: Supplementary file 1 [file ijms-22-12963-s001.zip › KovacsM_Fig legend to Suppl. Figures_2021 10 31_sm.pdf]

Figure S1 (a) Photo of the uncropped and unmodified Ponceau-stained membrane used later for the detection of angiotensin II type 1 receptor (AT1R) and GAPDH in the 1-week RIHD model. The efficacy of the transfer of proteins onto a nitrocellulose membrane was checked using Ponceau staining. Images were captured by the camera of an Apple iPhone10. (b) Unmodified Western blot images of angiotensin II type 1 receptor (AT1R) and (c) GAPDH in the 1-week RIHD model. Scanned images were captured with the Odyssey CLx machine and exported with Image Studio 5.2.5 software. Different parts of the same membrane are divided by black lines. The membrane was physically cut in the middle of the molecular weight marker at lane 17 before scanning. Cropped images were used in Fig. 5b. Ctrl: control group, RT: radiotherapy group (50 Gy), RT Los: RT plus losartan irradiated group, MW: molecular weight marker, NC: negative control, L: left, R: right.

Figure S2. (a) Photo of the uncropped and unmodified Ponceau-stained membrane used later for the detection of angiotensin II type 1 receptor (AT1R) and GAPDH in the 3-week RIHD model. The efficacy of the transfer of proteins onto a nitrocellulose membrane was checked using Ponceau staining. Images were captured by the camera of an Apple iPhone10. (b) Unmodified Western blot images of angiotensin II type 1 receptor (AT1R) and (c) GAPDH in the 3-week RIHD model. Scanned images were captured with the Odyssey CLx machine and exported with Image Studio 5.2.5 software. Different parts of the same membrane are divided by black lines. The membrane was physically cut in the middle of the molecular weight marker at lane 17 before scanning. Cropped images were used in Fig. 5b. Ctrl: control group, RT: radiotherapy group (50 Gy), RT Los: RT plus losartan irradiated group, MW: molecular weight marker, NC: negative control, L: left, R: right.

Figure S3 (a) Photo of the uncropped and unmodified Ponceau-stained membrane used later for the detection of angiotensin II type 1 receptor (AT1R) and GAPDH in the 15-week RIHD model. The efficacy of the transfer of proteins onto a nitrocellulose membrane was checked using Ponceau staining. Images were captured by the camera of an Apple iPhone10. (b) Unmodified Western blot images of angiotensin II type 1 receptor (AT1R) and (c) GAPDH in the 15-week RIHD model. Scanned images were captured with the Odyssey CLx machine and exported with Image Studio 5.2.5 software. Different parts of the same membrane are divided by black lines. The membrane was physically cut in the middle of the molecular weight marker at lane 17 before scanning. Cropped images were used in Fig. 5b. Ctrl: control group, RT: radiotherapy group (50 Gy), RT Los: RT plus losartan irradiated group, MW: molecular weight marker, NC: negative control, L: left, R: right.

Figure S4 (a) Photo of the uncropped and unmodified Ponceau-stained membrane used later for the detection of angiotensin II type 2 receptor (AT2R), TGF-β receptor II (TGF-βRII) and GAPDH in the 1-week RIHD model. The efficacy of the transfer of proteins onto a nitrocellulose membrane was checked using Ponceau staining. Images were captured by the camera of an Apple iPhone10. (b) Unmodified Western blot images of angiotensin II type 2 receptor (AT2R), TGF-β receptor II (TGF-βRII) and GAPDH in the 1-week RIHD model. Scanned images were captured with the Odyssey CLx machine and exported with Image Studio 5.2.5 software. Different parts of the same membrane are divided by black lines. The membrane was physically cut in the middle of the molecular weight marker at lane 17 before scanning. Cropped images were used in Fig. 5c and 5e. Ctrl: control group, RT: radiotherapy group (50 Gy), RT Los: RT plus losartan irradiated group, MW: molecular weight marker, NC: negative control, L: left, R: right.

Figure S5 (a) Photo of the uncropped and unmodified Ponceau-stained membrane used later for the detection of angiotensin II type 2 receptor (AT2R), TGF-β receptor II (TGF-βRII) and GAPDH in the 3-week RIHD model. The efficacy of the transfer of proteins onto a nitrocellulose membrane was checked using Ponceau staining. Images were captured by the camera of an Apple iPhone10. (b) Unmodified Western blot images of angiotensin II type 2 receptor (AT2R), TGF-β receptor II (TGF-βRII) and GAPDH

in the 3-week RIHD model. Scanned images were captured with the Odyssey CLx machine and exported with Image Studio 5.2.5 software. Different parts of the same membrane are divided by black lines. The membrane was physically cut in the middle of the molecular weight marker at lane 17 before scanning. Cropped images were used in Fig. 5c and 5e. Ctrl: control group, RT: radiotherapy group (50 Gy), RT Los: RT plus losartan irradiated group, MW: molecular weight marker, NC: negative control, L: left, R: right.

Figure S6 (a) Photo of the uncropped and unmodified Ponceau-stained membrane used later for the detection of angiotensin II type 2 receptor (AT2R), TGF-β receptor II and GAPDH in the 15-week RIHD model. The efficacy of the transfer of proteins onto a nitrocellulose membrane was checked using Ponceau staining. Images were captured by the camera of an Apple iPhone10. (b) Unmodified Western blot images of angiotensin II type 2 receptor (AT2R), TGF-β receptor II (TGF-βRII) and (c) GAPDH in the 15-week RIHD model. Scanned images were captured with the Odyssey CLx machine and exported with Image Studio 5.2.5 software. Different parts of the same membrane are divided by black lines. The membrane was physically cut in the middle of the molecular weight marker at lane 17 before scanning. Cropped images were used in Fig. 5c and 5e. Ctrl: control group, RT: radiotherapy group (50 Gy), RT Los: RT plus losartan irradiated group, MW: molecular weight marker, NC: negative control, L: left, R: right.

Figure S7 (a) Photo of the uncropped and unmodified Ponceau-stained membrane used later for the detection of SMAD2/3 and GAPDH in the 1-week RIHD model. The efficacy of the transfer of proteins onto a nitrocellulose membrane was checked using Ponceau staining. Images were captured by the camera of an Apple iPhone10. (b) Unmodified Western blot images of SMAD2/3 and (c) GAPDH in the 1-week RIHD model. Scanned images were captured with the Odyssey CLx machine and exported with Image Studio 5.2.5 software. Different parts of the same membrane are divided by black lines. The membrane was physically cut in the middle of the molecular weight marker at lane 17 before scanning. Cropped images were used in Fig. 5f. Ctrl: control group, RT: radiotherapy group (50 Gy), RT Los: RT plus losartan irradiated group, MW: molecular weight marker, NC: negative control, L: left, R: right.

Figure S8 (a) Photo of the uncropped and unmodified Ponceau-stained membrane used later for the detection of SMAD2/3 and GAPDH in the 3-week RIHD model. The efficacy of the transfer of proteins onto a nitrocellulose membrane was checked using Ponceau staining. Images were captured by the camera of an Apple iPhone10. (b) Unmodified Western blot images of SMAD2/3 and (c) GAPDH in the 3-week RIHD model. Scanned images were captured with the Odyssey CLx machine and exported with Image Studio 5.2.5 software. Different parts of the same membrane are divided by black lines. The membrane was physically cut in the middle of the molecular weight marker at lane 17 before scanning. Cropped images were used in Fig. 5f. Ctrl: control group, RT: radiotherapy group (50 Gy), RT Los: RT plus losartan irradiated group, MW: molecular weight marker, NC: negative control, L: left, R: right.

Figure S9 (a) Photo of the uncropped and unmodified Ponceau-stained membrane used later for the detection of SMAD2/3 and GAPDH in the 15-week RIHD model. The efficacy of the transfer of proteins onto a nitrocellulose membrane was checked using Ponceau staining. Images were captured by the camera of an Apple iPhone10. (b) Unmodified Western blot images of SMAD2/3 and (c) GAPDH in the 3-week RIHD model. Scanned images were captured with the Odyssey CLx machine and exported with Image Studio 5.2.5 software. Different parts of the same membrane are divided by black lines. The membrane was physically cut in the middle of the molecular weight marker at lane 17 before scanning. Cropped images were used in Fig. 5f. Ctrl: control group, RT: radiotherapy group (50 Gy), RT Los: RT plus losartan irradiated group, MW: molecular weight marker, NC: negative control, L: left, R: right.

Figure S10 The effects of losartan on the expression of phospho(p)-ERK1,2 and total (t) ERK1,2 in the left ventricle at weeks 1, 3, and 15. Left ventricular expression and cropped representative images of (a)

tERK1, (b) tERK2, (c) pERK1, and (d) pERK2 normalized to GAPDH. Values are presented as mean±S.E.M., *p< 0.05 vs. control group, #p<0.05 vs. RT group (n=6–7, One-Way ANOVA, Holm-Sidak post hoc test). Ctrl: control group, RT: radiotherapy group (50 Gy), RT Los: radiotherapy plus losartan group. Images were captured with the Odyssey CLx machine and exported with Image Studio 5.2.5 software. The full-length Western blots are presented in the Supplementary Figures S12-17.

Figure S11 The effects of losartan on the expression of phospho(p)-AKT, total (t) AKT, phospho(p)-STAT3 and total (t) STAT3 in the left ventricle at weeks 1, 3, and 15. Left ventricular expression and cropped representative images of (a) tAKT, (b) pAKT, (c) tSTAT3, and (d) pSTAT3 normalized to GAPDH. Values are presented as mean±S.E.M., *p< 0.05 vs. control group, #p<0.05 vs. RT group (n=6–7, One-Way ANOVA, Holm-Sidak post hoc test). Ctrl: control group, RT: radiotherapy group (50 Gy), RT Los: radiotherapy plus losartan group. Images were captured with the Odyssey CLx machine and exported with Image Studio 5.2.5 software. The full-length Western blots are presented in the Supplementary Material S12-17.

Figure S12 (a) Photo of the uncropped and unmodified Ponceau-stained membrane used later for the detection of pSTAT, pAKT, pERK1, pERK2 and GAPDH in the 1-week RIHD model. The efficacy of the transfer of proteins onto a nitrocellulose membrane was checked using Ponceau staining. Images were captured by the camera of an Apple iPhone10. (b) Unmodified Western blot images of pSTAT, pAKT, pERK1, pERK2 and (c) GAPDH in the 1-week RIHD model. Scanned images were captured with the Odyssey CLx machine and exported with Image Studio 5.2.5 software. Different parts of the same membrane are divided by black lines. The membrane was physically cut in the middle of the molecular weight marker at lane 17 before scanning. Cropped images were used in Fig. 6a-6d, Fig S10 and S11. Ctrl: control group, RT: radiotherapy group (50 Gy), RT Los: RT plus losartan irradiated group, MW: molecular weight marker, NC: negative control, L: left, R: right

Figure S13 (a) Photo of the uncropped and unmodified Ponceau-stained membrane used later for the detection of pSTAT, pAKT, pERK1, pERK2 and GAPDH in the 3-week RIHD model. The efficacy of the transfer of proteins onto a nitrocellulose membrane was checked using Ponceau staining. Images were captured by the camera of an Apple iPhone10. (b) Unmodified Western blot images of pSTAT, pAKT, pERK1, pERK2 and (c) GAPDH in the 3-week RIHD model. Scanned images were captured with the Odyssey CLx machine and exported with Image Studio 5.2.5 software. Different parts of the same membrane are divided by black lines. The membrane was physically cut in the middle of the molecular weight marker at lane 17 before scanning. Cropped images were used in Fig. 6a-6d, Fig S1 and S2. Ctrl: control group, RT: radiotherapy group (50 Gy), RT Los: RT plus losartan irradiated group, MW: molecular weight marker, NC: negative control, L: left, R: right

Figure S14 (a) Photo of the uncropped and unmodified Ponceau-stained membrane used later for the detection of pSTAT, pAKT, pERK1, pERK2 and GAPDH in the 15-week RIHD model. The efficacy of the transfer of proteins onto a nitrocellulose membrane was checked using Ponceau staining. Images were captured by the camera of an Apple iPhone10. (b) Unmodified Western blot images of pSTAT, pAKT, pERK1, pERK2 and (c) GAPDH in the 15-week RIHD model. Scanned images were captured with the Odyssey CLx machine and exported with Image Studio 5.2.5 software. Different parts of the same membrane are divided by black lines. The membrane was physically cut in the middle of the molecular weight marker at lane 17 before scanning. Cropped images were used in Fig. 6a-6d, Fig S10 and S11. Ctrl: control group, RT: radiotherapy group (50 Gy), RT Los: RT plus losartan irradiated group, MW: molecular weight marker, NC: negative control, L: left, R: right

Figure S15 (a) Photo of the uncropped and unmodified Ponceau-stained membrane used later for the detection of tSTAT, tAKT, tERK1, tERK2 and GAPDH in the 1-week RIHD model. The efficacy of the transfer of proteins onto a nitrocellulose membrane was checked using Ponceau staining. Images were

captured by the camera of an Apple iPhone10. (b) Unmodified Western blot images of tSTAT, tAKT, tERK1, tERK2 and (c) GAPDH in the 1-week RIHD model. Scanned images were captured with the Odyssey CLx machine and exported with Image Studio 5.2.5 software. Different parts of the same membrane are divided by black lines. The membrane was physically cut in the middle of the molecular weight marker at lane 17 before scanning. Cropped images were used in Fig. 6a-6d, Fig. S10 and S11. Ctrl: control group, RT: radiotherapy group (50 Gy), RT Los: RT plus losartan irradiated group, MW: molecular weight marker, NC: negative control, L: left, R: right

Figure S16 (a) Photo of the uncropped and unmodified Ponceau-stained membrane used later for the detection of tSTAT, tAKT, tERK1, tERK2 and GAPDH in the 3-week RIHD model. The efficacy of the transfer of proteins onto a nitrocellulose membrane was checked using Ponceau staining. Images were captured by the camera of an Apple iPhone10. (b) Unmodified Western blot images of tSTAT, tAKT, tERK1, tERK2 and (c) GAPDH in the 3-week RIHD model. Scanned images were captured with the Odyssey CLx machine and exported with Image Studio 5.2.5 software. Different parts of the same membrane are divided by black lines. The membrane was physically cut in the middle of the molecular weight marker at lane 17 before scanning. Cropped images were used in Fig. 6a-6d, Fig. S10 and S11. Ctrl: control group, RT: radiotherapy group (50 Gy), RT Los: RT plus losartan irradiated group, MW: molecular weight marker, NC: negative control, L: left, R: right

Figure S17 (a) Photo of the uncropped and unmodified Ponceau-stained membrane used later for the detection of tSTAT, tAKT, tERK1, tERK2 and GAPDH in the 15-week RIHD model. The efficacy of the transfer of proteins onto a nitrocellulose membrane was checked using Ponceau staining. Images were captured by the camera of an Apple iPhone10. (b) Unmodified Western blot images of tSTAT, tAKT, tERK1, tERK2 and (c) GAPDH in the 15-week RIHD model. Scanned images were captured with the Odyssey CLx machine and exported with Image Studio 5.2.5 software. Different parts of the same membrane are divided by black lines. The membrane was physically cut in the middle of the molecular weight marker at lane 17 before scanning. Cropped images were used in Fig. 6a-6d, Fig. S10 and S11. Ctrl: control group, RT: radiotherapy group (50 Gy), RT Los: RT plus losartan irradiated group, MW: molecular weight marker, NC: negative control, L: left, R: right
