# Supplementary material for: Investigation of the Antihypertrophic and Antifibrotic Effects of Losartan in a Rat Model of Radiation-Induced Heart Disease
Source: Int J Mol Sci. 2021 Nov 30;22(23):12963. doi: 10.3390/ijms222312963 (PMC8657420; doi:10.3390/ijms222312963)
Supplement: Supplementary file 1 [file ijms-22-12963-s001.zip › KovacsM_Suppl Figures_2021 okt 30_sm.pptx]

## Slide 1
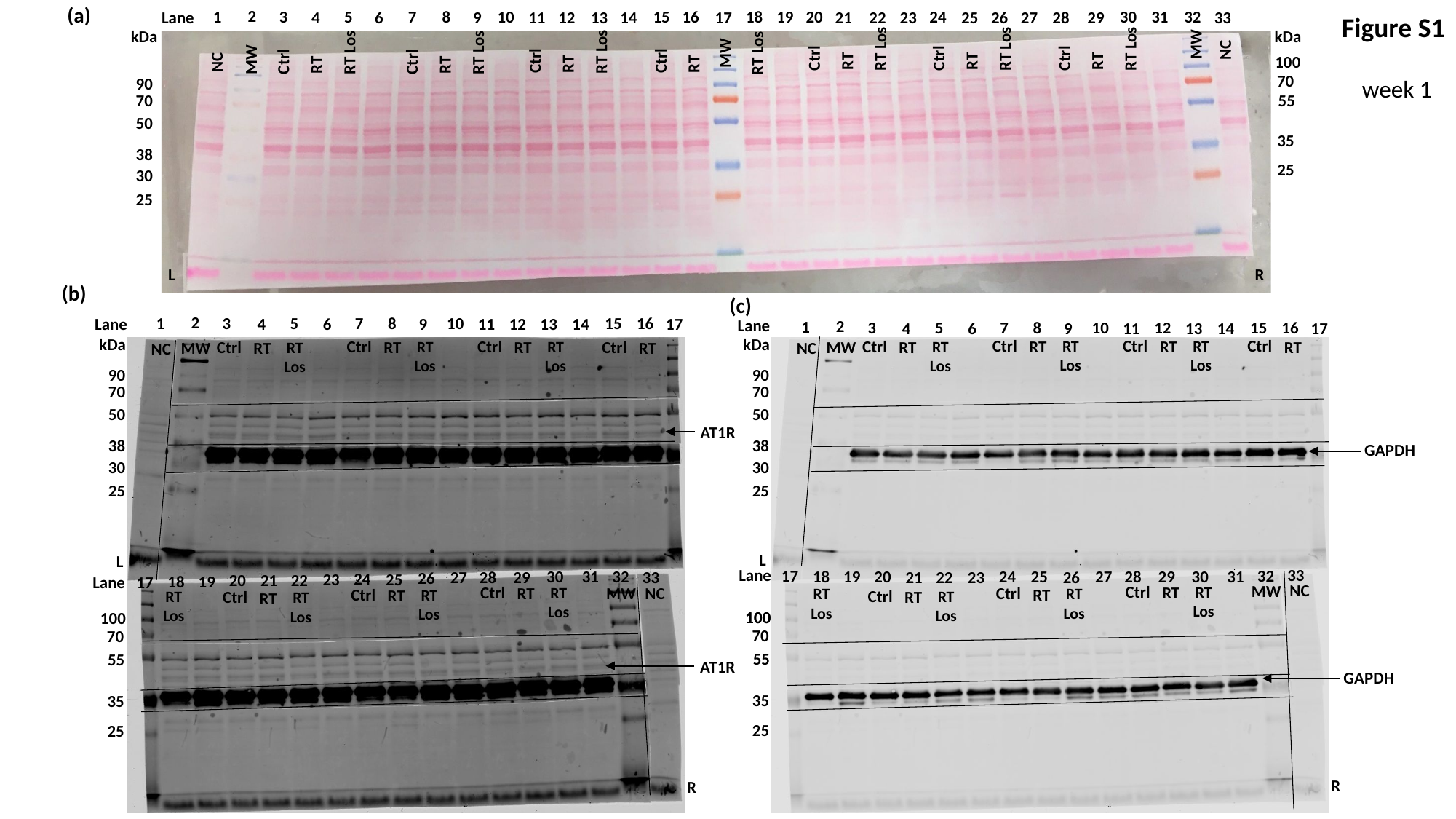

2
(a)
1
3
19
8
24
30
31
32
7
16
10
20
5
15
18
Lane
12
21
22
23
29
4
6
14
17
11
9
13
25
26
27
28
33
Figure S1
kDa
kDa
MW
RT Los
RT Los
RT Los
NC
RT Los
RT Los
RT Los
MW
RT Los
Ctrl
Ctrl
Ctrl
MW
Ctrl
Ctrl
100
RT
RT
RT
Ctrl
Ctrl
NC
RT
RT
RT
RT
70
90
week 1
55
70
50
35
38
25
30
25
L
R
(b)
(c)
2
1
3
7
16
10
5
15
8
Lane
12
4
6
14
17
11
9
13
Lane
2
1
3
7
16
10
5
15
8
12
4
6
14
17
11
9
13
kDa
kDa
Ctrl
Ctrl
Ctrl
Ctrl
Ctrl
Ctrl
RT
RT Los
RT
RT Los
Ctrl
RT
Ctrl
RT
RT Los
RT
RT Los
RT
RT Los
MW
RT
NC
RT
RT Los
MW
NC
90
90
70
70
50
50
AT1R
38
38
GAPDH
30
30
25
25
L
L
33
Lane
32
17
25
29
28
27
30
31
32
19
24
30
18
20
31
21
22
23
28
26
27
29
33
26
24
23
25
20
21
22
19
18
Lane
17
NC
MW
Ctrl
RT
RT Los
Ctrl
MW
NC
RT
RT Los
Ctrl
RT Los
RT
RT Los
Ctrl
RT
RT Los
Ctrl
RT Los
RT
RT Los
Ctrl
RT
RT Los
100
100
100
70
70
55
55
AT1R
GAPDH
35
35
25
25
R
R

## Slide 2
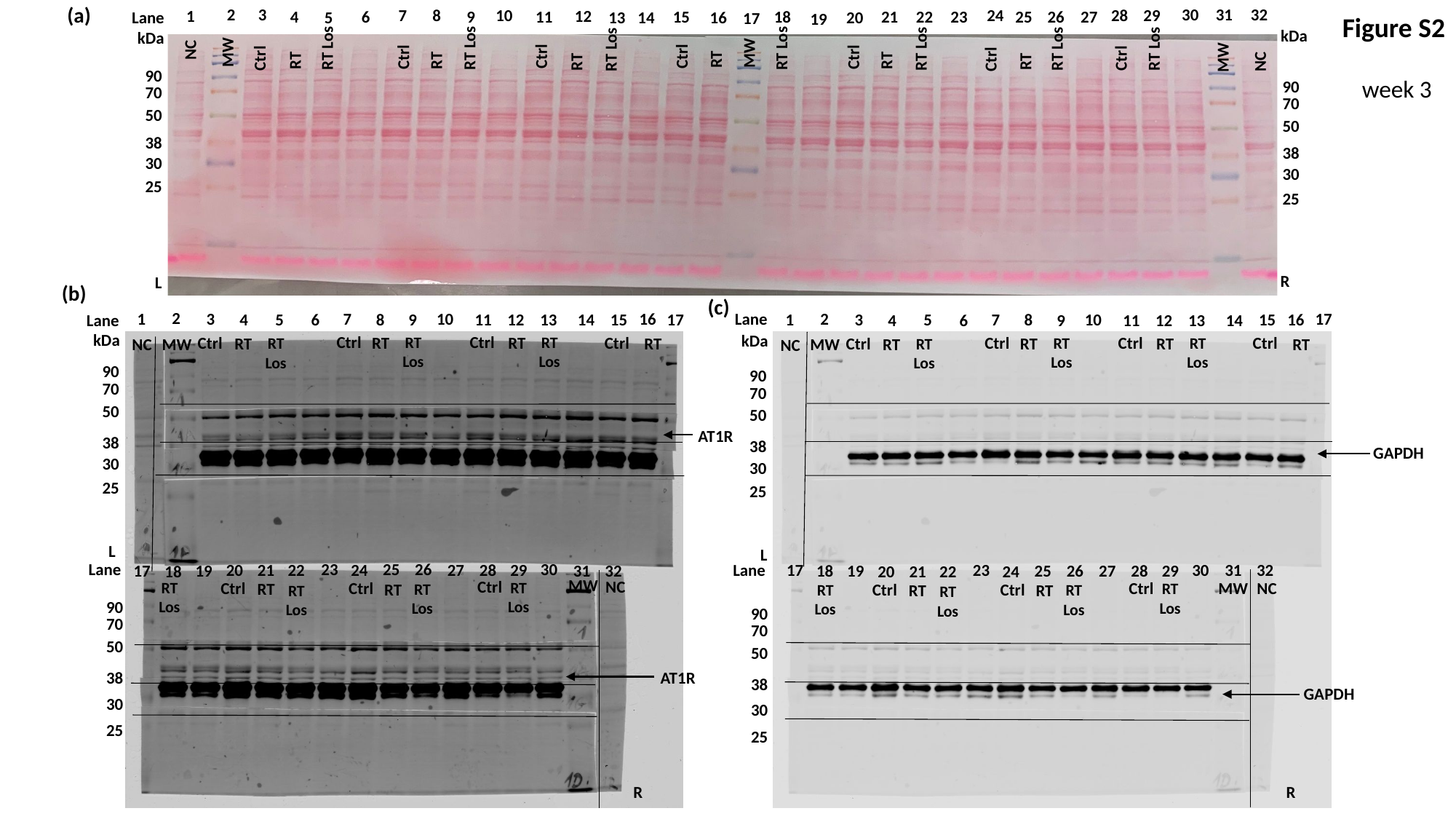

12
(a)
3
32
30
2
31
29
28
8
10
24
7
1
23
6
4
21
11
9
22
25
26
27
18
15
5
16
20
Lane
14
13
17
19
Figure S2
kDa
kDa
RT Los
RT Los
RT Los
RT Los
RT Los
RT Los
RT Los
NC
MW
MW
Ctrl
Ctrl
Ctrl
Ctrl
MW
Ctrl
Ctrl
RT
Ctrl
RT
RT
RT
RT
NC
RT
90
week 3
90
70
70
50
50
38
38
30
30
25
25
R
L
(b)
(c)
2
2
1
3
17
7
16
10
Lane
5
15
8
1
12
3
7
4
6
14
17
16
10
5
15
11
9
13
8
Lane
12
4
6
14
11
9
13
kDa
kDa
Ctrl
Ctrl
Ctrl
Ctrl
Ctrl
Ctrl
RT
RT Los
RT
RT Los
Ctrl
RT
Ctrl
RT
RT Los
RT
RT Los
RT
RT Los
MW
RT
NC
RT
RT Los
MW
NC
90
90
70
70
50
50
AT1R
38
38
GAPDH
30
30
25
25
L
L
Lane
23
30
26
25
28
27
29
24
20
32
Lane
17
21
22
23
30
31
17
26
25
32
31
18
19
28
27
29
19
24
20
21
22
18
MW
Ctrl
NC
Ctrl
MW
RT Los
NC
RT Los
Ctrl
Ctrl
RT
RT Los
Ctrl
RT
Ctrl
RT Los
RT
RT Los
RT
RT Los
RT Los
RT Los
90
90
70
70
50
50
38
AT1R
38
GAPDH
30
30
25
25
R
R

## Slide 3
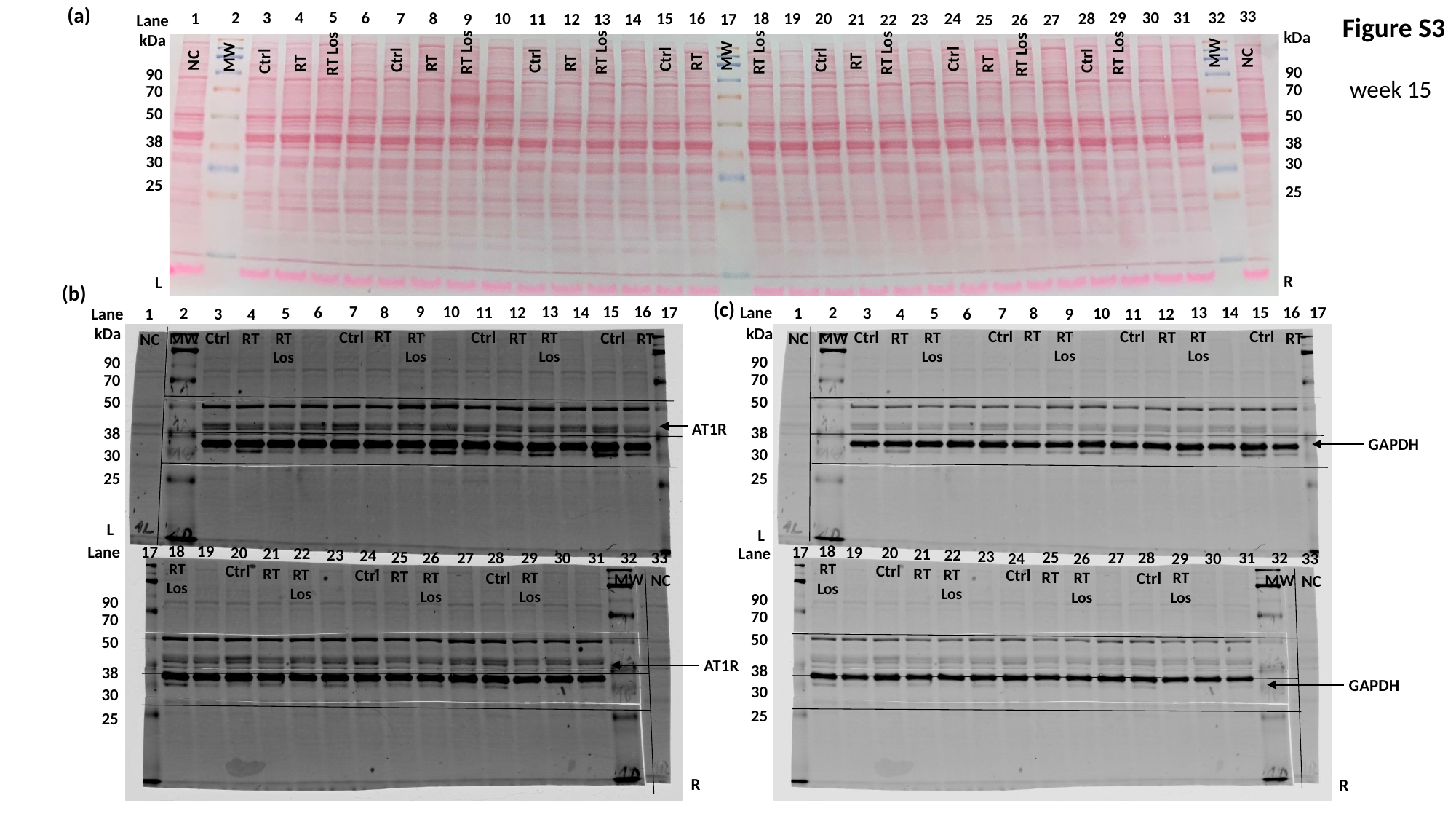

(a)
33
5
3
2
6
30
31
32
4
29
19
15
16
18
28
20
8
10
24
1
7
12
14
9
13
17
23
11
21
25
26
22
27
Lane
Figure S3
kDa
kDa
RT Los
RT Los
RT Los
MW
RT Los
RT Los
RT Los
RT Los
MW
MW
NC
Ctrl
Ctrl
Ctrl
Ctrl
NC
Ctrl
Ctrl
Ctrl
RT
RT
RT
RT
RT
RT
90
90
week 15
70
70
50
50
38
38
30
30
25
25
R
L
(b)
(c)
13
7
9
10
15
16
8
16
2
11
12
14
17
15
6
14
17
13
Lane
1
2
3
7
10
5
5
8
Lane
1
12
3
4
6
11
9
4
kDa
kDa
RT
Ctrl
Ctrl
RT
Ctrl
Ctrl
Ctrl
Ctrl
RT Los
RT
RT Los
Ctrl
RT
Ctrl
RT Los
RT
RT Los
RT
RT Los
MW
RT
NC
RT
RT Los
MW
NC
90
90
70
70
50
50
AT1R
38
38
GAPDH
30
30
25
25
L
L
19
18
18
17
Lane
17
20
19
20
Lane
21
22
21
23
22
24
23
25
25
27
32
28
31
27
26
29
30
33
32
28
31
24
26
29
30
33
RT Los
RT Los
Ctrl
Ctrl
RT
RT
RT Los
Ctrl
RT Los
Ctrl
RT
RT
Ctrl
Ctrl
RT Los
RT Los
RT Los
RT Los
MW
MW
NC
NC
90
90
70
70
50
50
AT1R
38
38
GAPDH
30
30
25
25
R
R

## Slide 4
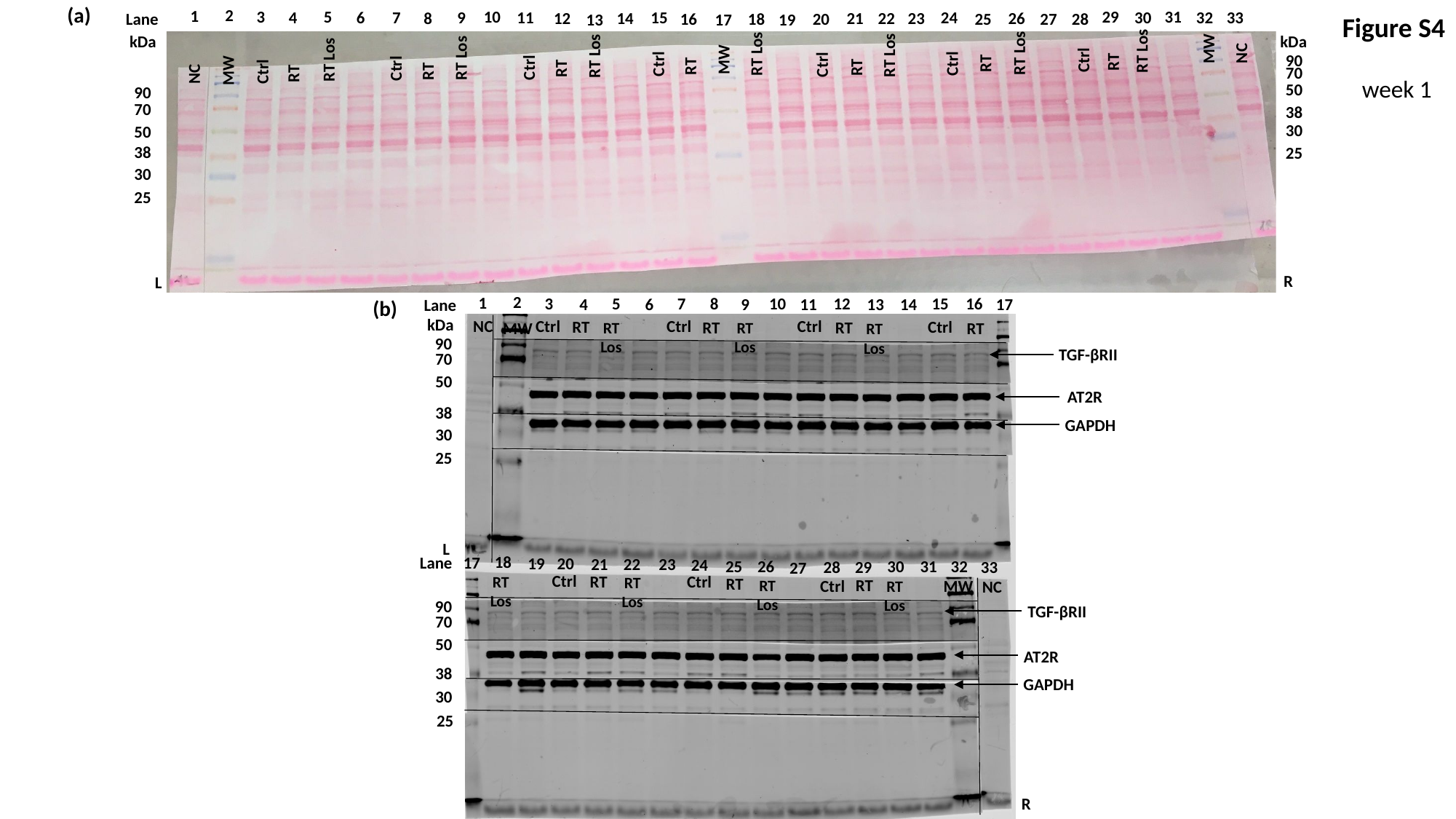

(a)
2
1
3
31
10
5
29
4
6
11
7
9
33
30
32
24
15
8
22
23
12
14
21
26
Lane
18
25
27
28
20
16
13
17
19
Figure S4
kDa
kDa
MW
RT Los
RT Los
NC
RT Los
RT Los
RT Los
RT Los
MW
Ctrl
90
RT Los
RT
RT
Ctrl
Ctrl
Ctrl
RT
Ctrl
RT
Ctrl
RT
MW
RT
Ctrl
70
RT
NC
week 1
50
90
70
38
30
50
38
25
30
25
R
L
2
1
3
7
16
10
5
15
8
12
4
6
14
17
11
9
13
Lane
(b)
kDa
NC
Ctrl
Ctrl
Ctrl
Ctrl
RT
RT
RT
RT
MW
RT Los
RT Los
RT Los
90
TGF-βRII
70
50
AT2R
38
GAPDH
30
25
L
18
Lane
17
19
20
21
22
23
24
25
31
30
32
26
28
29
33
27
Ctrl
RT
Ctrl
RT Los
RT Los
RT
RT
Ctrl
MW
RT Los
RT Los
NC
90
TGF-βRII
70
50
AT2R
38
GAPDH
30
25
R

## Slide 5
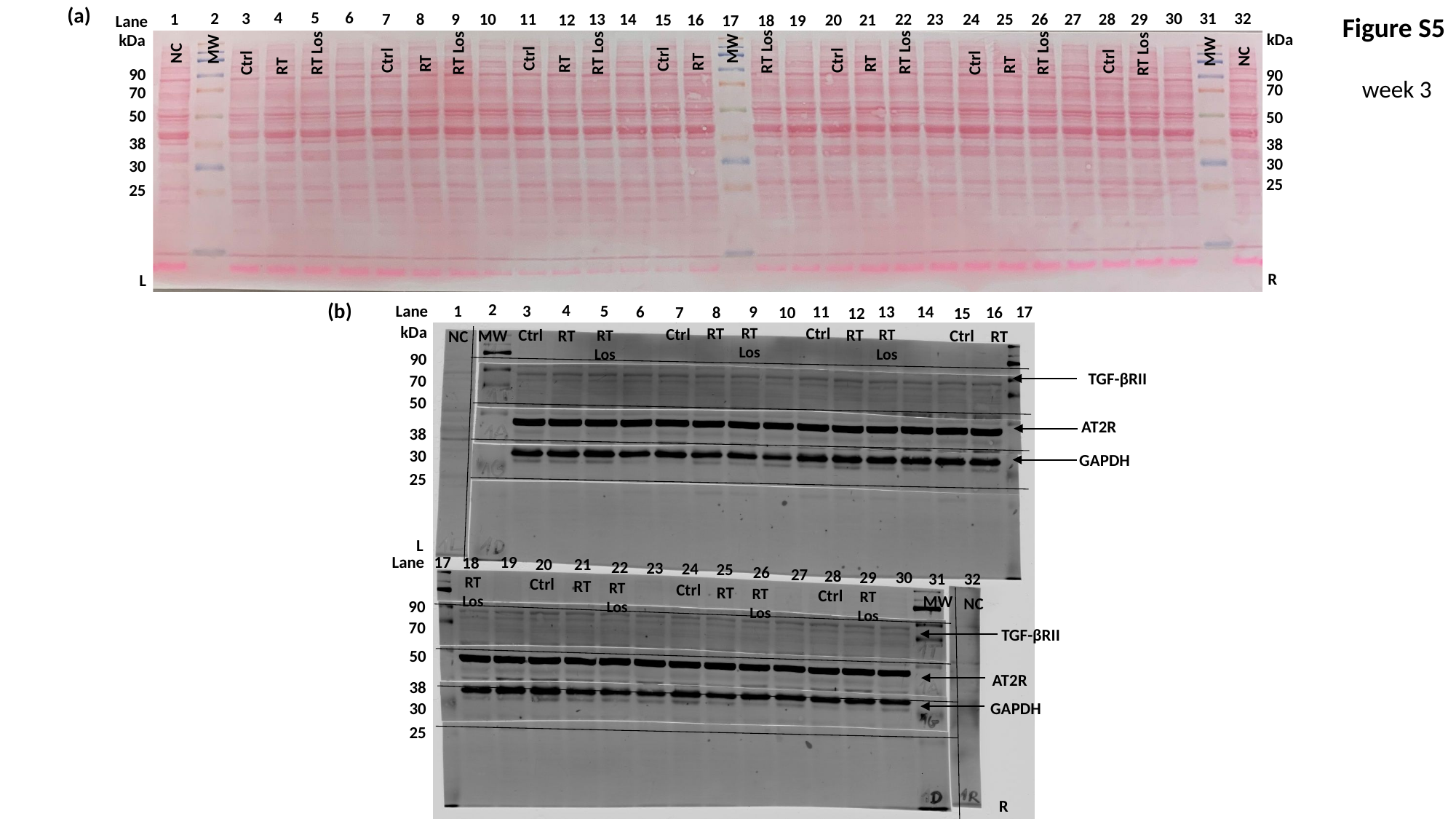

(a)
5
6
4
3
32
30
2
31
23
29
28
22
11
8
9
10
14
24
25
26
27
7
1
13
12
15
16
20
21
19
17
18
Figure S5
Lane
kDa
kDa
MW
MW
MW
RT Los
RT Los
RT Los
RT Los
RT Los
NC
RT Los
RT Los
NC
Ctrl
Ctrl
Ctrl
Ctrl
Ctrl
RT
Ctrl
Ctrl
RT
RT
RT
RT
RT
90
90
week 3
70
70
50
50
38
38
30
30
25
25
R
L
(b)
2
4
Lane
1
3
5
6
14
17
13
9
11
7
8
10
16
12
15
kDa
RT
RT Los
Ctrl
Ctrl
Ctrl
RT
RT Los
RT Los
RT
MW
Ctrl
NC
RT
90
TGF-βRII
70
50
AT2R
38
30
GAPDH
25
L
17
Lane
19
18
20
21
22
23
24
25
26
27
28
29
30
31
32
RT Los
Ctrl
RT
RT Los
Ctrl
RT
RT Los
Ctrl
RT Los
MW
NC
90
70
TGF-βRII
50
AT2R
38
30
GAPDH
25
R

## Slide 6
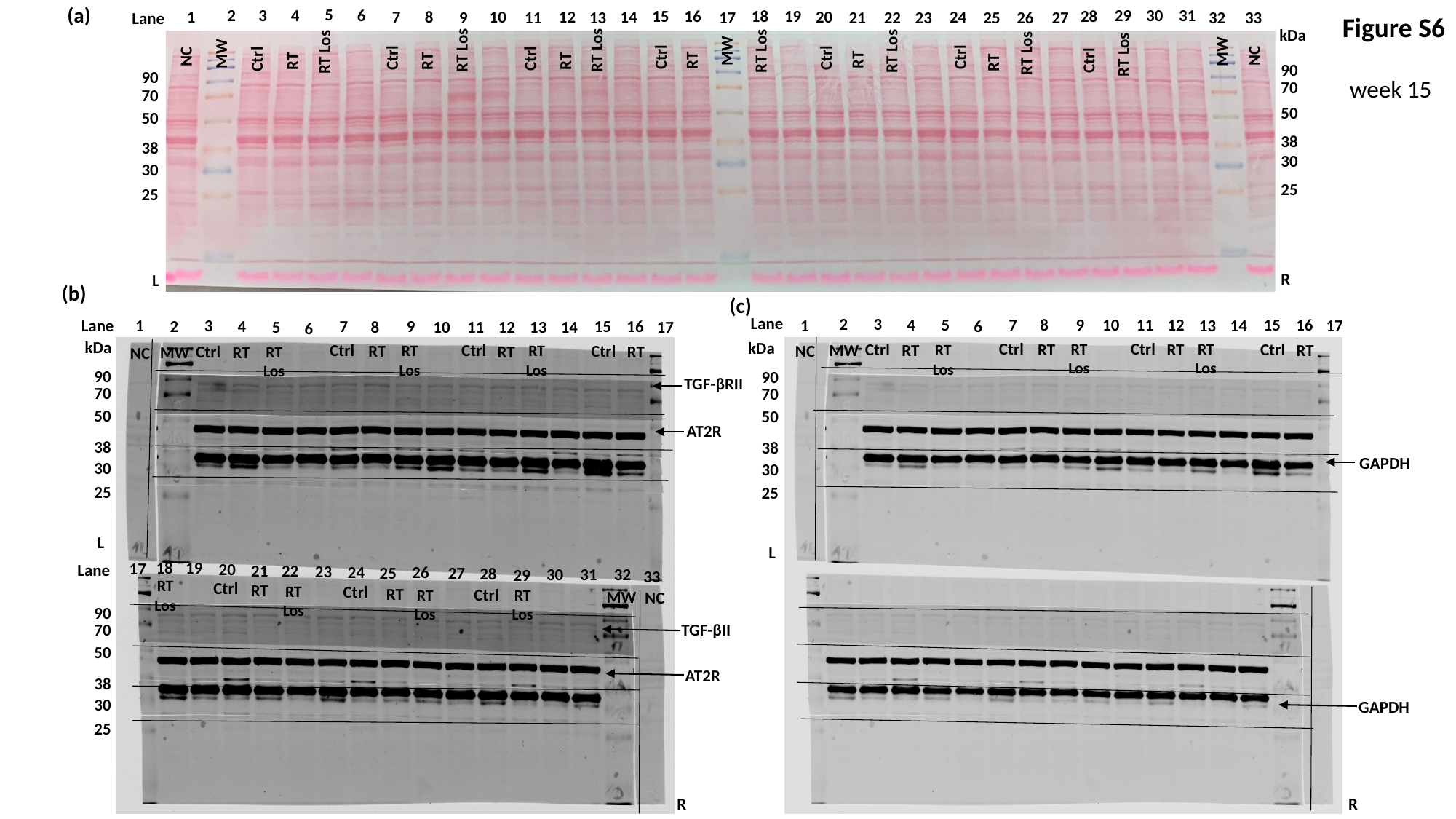

(a)
5
3
2
6
30
31
4
29
19
15
16
18
28
20
8
10
24
1
7
12
14
9
13
17
23
11
21
32
33
25
26
22
27
Lane
Figure S6
kDa
RT Los
RT Los
MW
RT Los
RT Los
MW
RT Los
RT Los
MW
RT Los
NC
NC
Ctrl
Ctrl
Ctrl
Ctrl
Ctrl
Ctrl
RT
RT
Ctrl
RT
RT
RT
RT
90
90
week 15
70
70
50
50
38
38
30
30
25
25
R
L
(b)
(c)
Lane
3
2
15
16
4
7
9
10
11
5
8
12
17
1
14
13
Lane
1
3
2
15
16
6
4
7
9
10
11
5
8
12
17
14
13
6
kDa
kDa
Ctrl
Ctrl
RT
Ctrl
RT Los
RT Los
Ctrl
RT
RT
RT
RT Los
Ctrl
Ctrl
MW
NC
RT
Ctrl
RT Los
RT Los
Ctrl
RT
RT
RT
RT Los
MW
NC
90
90
TGF-βRII
70
70
50
50
AT2R
38
38
GAPDH
30
30
25
25
L
L
19
18
17
20
Lane
21
22
23
26
24
25
27
28
30
31
32
29
33
RT Los
Ctrl
RT
RT Los
Ctrl
RT
Ctrl
RT Los
RT Los
MW
NC
90
70
TGF-βII
50
AT2R
38
30
GAPDH
25
R
R

## Slide 7
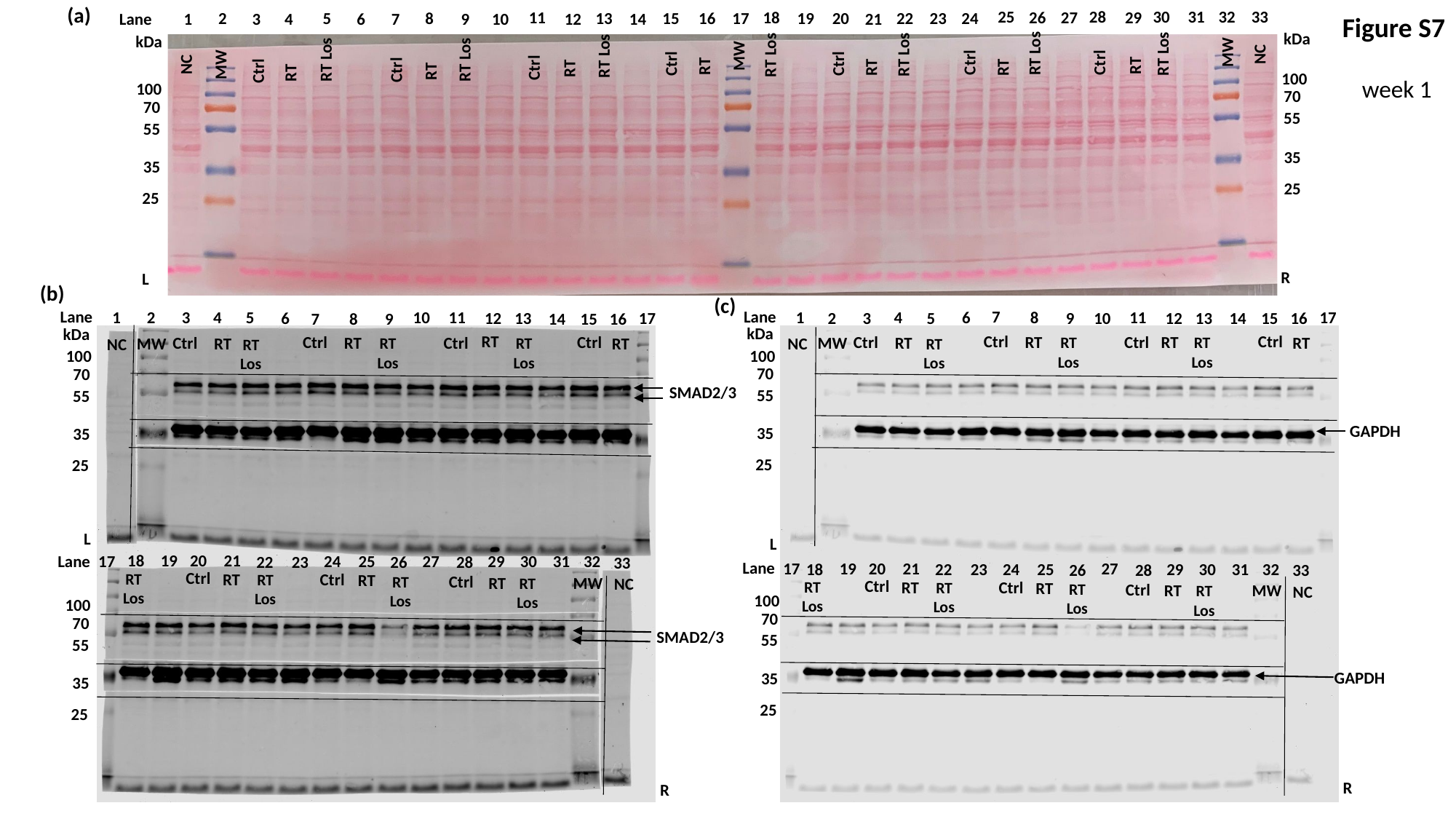

(a)
25
28
32
33
30
31
27
29
26
11
18
13
17
15
8
2
5
19
16
20
23
24
22
21
6
12
14
4
10
1
3
7
9
Lane
Figure S7
kDa
kDa
MW
RT Los
RT Los
RT Los
NC
RT Los
RT Los
MW
RT Los
RT Los
Ctrl
Ctrl
Ctrl
Ctrl
MW
NC
RT
RT
RT
RT
Ctrl
RT
Ctrl
Ctrl
RT
RT
100
week 1
100
70
70
55
55
35
35
25
25
R
L
(b)
(c)
Lane
Lane
7
8
4
1
11
3
4
1
11
2
10
5
6
17
3
13
9
12
2
10
5
14
15
16
6
17
12
13
9
14
7
15
16
8
kDa
kDa
Ctrl
RT
Ctrl
Ctrl
Ctrl
RT
Ctrl
RT
Ctrl
Ctrl
RT
RT
Ctrl
RT
MW
RT
RT Los
RT Los
NC
RT
MW
RT Los
RT Los
NC
RT Los
RT Los
100
100
70
70
SMAD2/3
55
55
GAPDH
35
35
25
25
L
L
19
20
18
21
17
27
29
Lane
24
25
31
30
32
22
23
26
28
33
Lane
19
20
21
17
27
29
24
25
31
18
30
32
22
23
26
28
33
Ctrl
RT
Ctrl
RT Los
RT Los
RT
Ctrl
RT Los
RT
MW
RT Los
NC
Ctrl
RT
Ctrl
RT Los
RT Los
RT
Ctrl
RT Los
RT
MW
RT Los
NC
100
100
70
70
SMAD2/3
55
55
GAPDH
35
35
25
25
R
R

## Slide 8
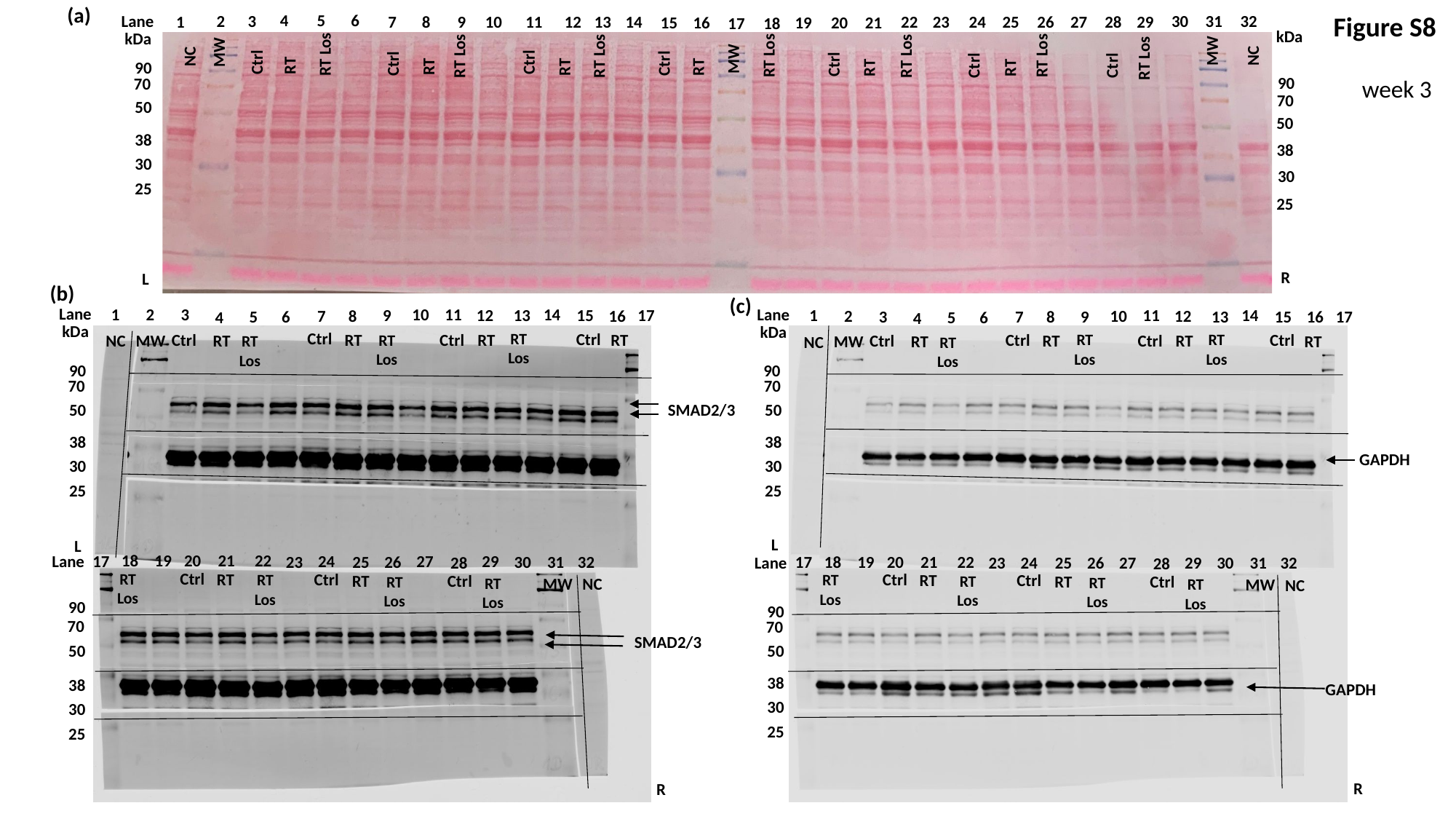

(a)
Figure S8
5
6
4
3
32
30
2
31
Lane
23
29
28
22
11
8
9
10
14
24
25
26
27
7
1
13
12
15
16
20
21
19
17
18
kDa
kDa
MW
MW
RT Los
RT Los
RT Los
RT Los
RT Los
NC
RT Los
NC
RT Los
MW
Ctrl
Ctrl
Ctrl
Ctrl
Ctrl
RT
Ctrl
Ctrl
RT
RT
RT
90
RT
RT
90
70
week 3
70
50
50
38
38
30
30
25
25
R
L
(b)
(c)
Lane
1
11
14
3
2
10
Lane
12
1
11
14
17
13
9
15
2
10
7
16
12
8
17
13
5
9
15
6
7
16
3
8
4
5
6
4
kDa
kDa
Ctrl
Ctrl
RT Los
Ctrl
Ctrl
RT
Ctrl
RT
Ctrl
RT
RT Los
RT Los
RT
Ctrl
RT
MW
RT Los
NC
Ctrl
RT
RT
RT
MW
RT Los
NC
RT Los
90
90
70
70
SMAD2/3
50
50
38
38
GAPDH
30
30
25
25
L
L
19
20
22
18
21
17
27
29
Lane
24
19
20
22
18
25
31
30
32
21
23
26
17
27
29
Lane
28
24
25
31
30
32
23
26
28
Ctrl
RT
Ctrl
Ctrl
RT Los
RT
Ctrl
RT Los
RT Los
RT
Ctrl
RT Los
RT
Ctrl
RT Los
RT Los
MW
RT Los
NC
MW
RT Los
NC
90
90
70
70
SMAD2/3
50
50
38
38
GAPDH
30
30
25
25
R
R

## Slide 9
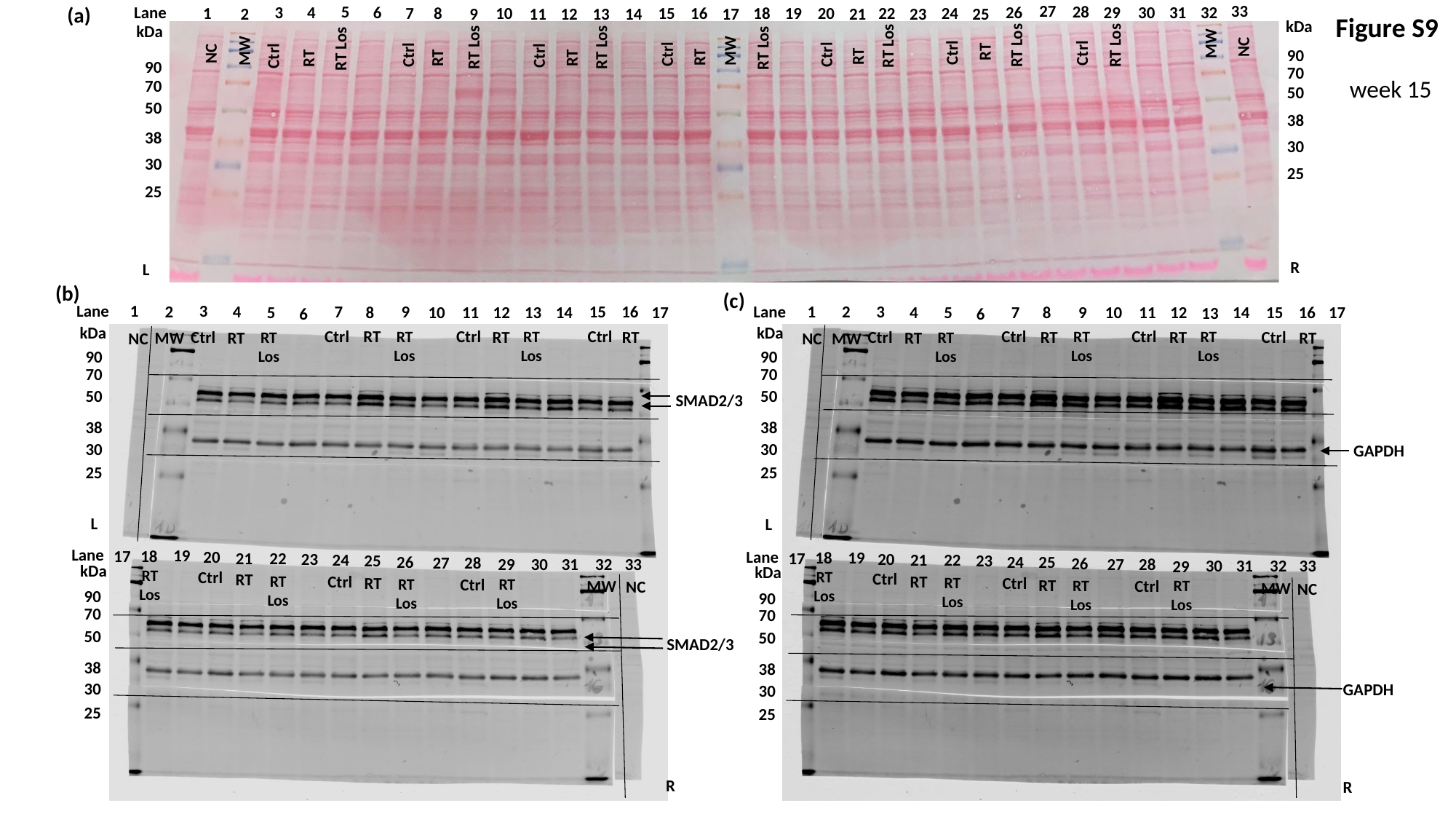

33
27
28
5
Lane
3
30
31
6
4
26
29
32
(a)
22
15
16
18
19
20
7
24
8
10
1
12
14
21
25
2
17
9
13
23
11
Figure S9
kDa
kDa
MW
RT Los
RT Los
RT Los
NC
RT Los
RT Los
RT Los
RT Los
MW
MW
RT
Ctrl
Ctrl
NC
Ctrl
Ctrl
Ctrl
90
Ctrl
Ctrl
RT
RT
RT
RT
RT
90
70
week 15
70
50
50
38
38
30
30
25
25
R
L
(b)
(c)
Lane
1
3
Lane
1
2
3
2
15
16
4
7
9
10
11
15
16
5
8
12
17
4
7
9
14
10
13
11
5
8
12
17
14
13
6
6
kDa
kDa
Ctrl
Ctrl
Ctrl
Ctrl
RT
Ctrl
RT Los
RT Los
RT
Ctrl
RT
Ctrl
RT Los
RT Los
Ctrl
RT
RT
RT
RT Los
RT
MW
RT
RT Los
NC
MW
NC
90
90
70
70
50
50
SMAD2/3
38
38
30
30
GAPDH
25
25
L
L
Lane
19
18
17
Lane
20
19
18
17
21
22
20
23
21
22
24
25
23
26
24
25
27
28
26
30
31
32
33
27
29
28
30
31
32
33
29
kDa
kDa
RT Los
Ctrl
RT Los
Ctrl
RT
RT Los
Ctrl
RT
RT
RT Los
Ctrl
Ctrl
RT Los
RT Los
RT
Ctrl
MW
RT Los
RT Los
NC
MW
NC
90
90
70
70
50
50
SMAD2/3
38
38
30
GAPDH
30
25
25
R
R

## Slide 10
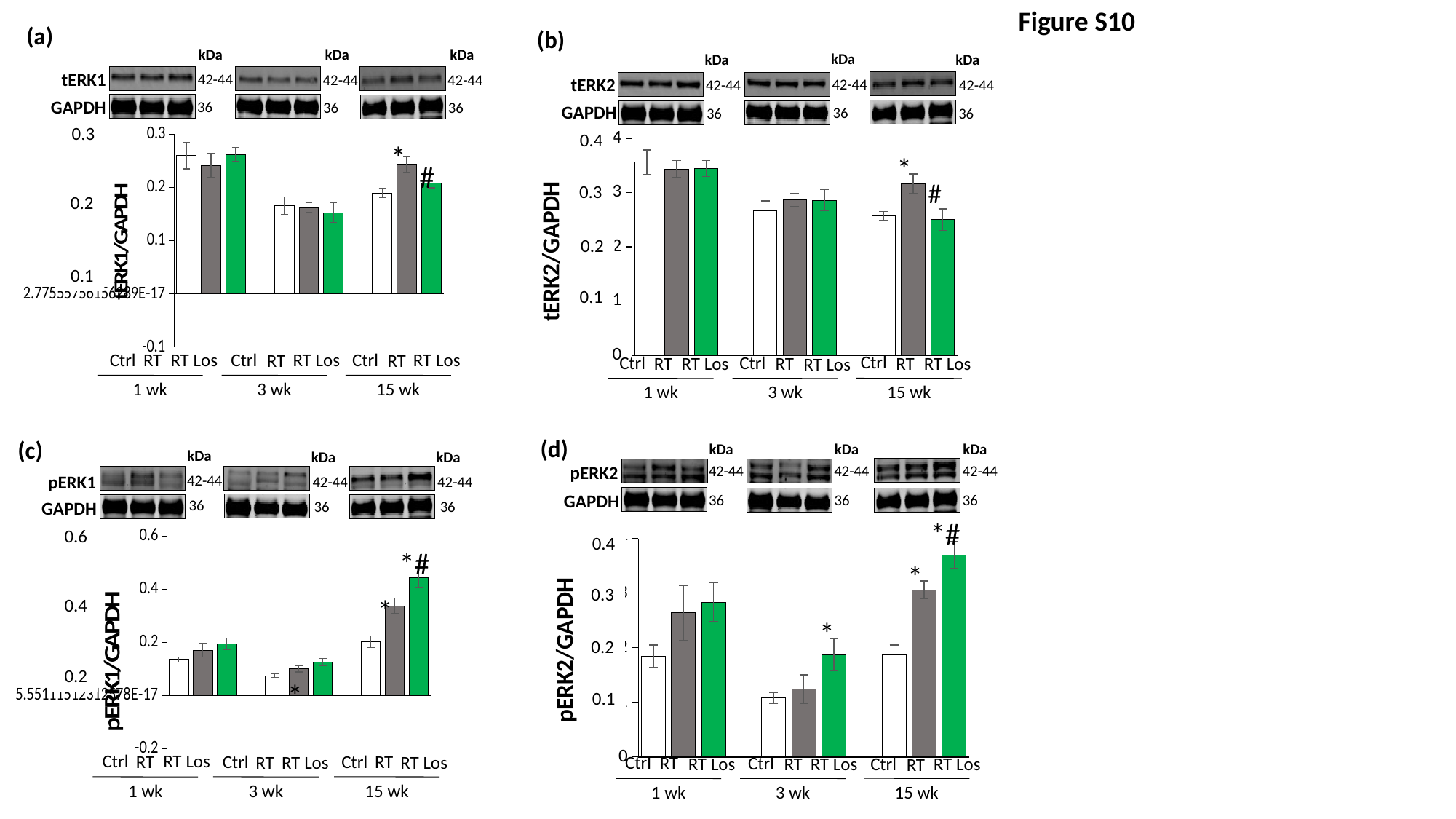

Figure S10
(a)
(b)
kDa
kDa
kDa
tERK1
42-44
42-44
42-44
GAPDH
36
36
36
### Chart
| Category | |
|---|---|
| Control | 0.25947690959840813 |
| RT | 0.24095718081203646 |
| RT Los | 0.2618412512280046 |
| | None |
| Control | 0.1656322431255947 |
| RT | 0.16212926996780885 |
| RT Los | 0.1525465574755613 |
| | None |
| Control | 0.18939892819690277 |
| RT | 0.2431445969342548 |
| RT Los | 0.20867197123968761 |*
#
0.3
0.2
0.1
RT Los
Ctrl
RT Los
Ctrl
RT Los
RT
Ctrl
RT
RT
15 wk
1 wk
3 wk
kDa
kDa
kDa
tERK2
42-44
42-44
42-44
GAPDH
36
36
36
0.4
0.3
0.2
0.1
Ctrl
Ctrl
Ctrl
RT Los
RT
RT
RT
RT Los
RT Los
15 wk
1 wk
3 wk
### Chart
| Category | |
|---|---|
| Control | 0.3560599212731742 |
| RT | 0.34340113772593034 |
| RT Los | 0.34425454153932034 |
| | None |
| Control | 0.2660226157846824 |
| RT | 0.28609066395043553 |
| RT Los | 0.28577135411509014 |
| | None |
| Control | 0.2567699151759411 |
| RT | 0.3164066871126279 |
| RT Los | 0.250126320711749 |*
#
(d)
(c)
kDa
kDa
kDa
kDa
kDa
kDa
pERK1
42-44
42-44
42-44
36
GAPDH
36
36
### Chart
| Category | |
|---|---|
| Control | 0.13458786721962504 |
| RT | 0.17009948368887454 |
| RT Los | 0.1940568634056509 |
| | None |
| Control | 0.07394583568121618 |
| RT | 0.09919900691750276 |
| RT Los | 0.12514930277672515 |
| | None |
| Control | 0.20109349016095915 |
| RT | 0.3371912534291018 |
| RT Los | 0.4418388261875389 |*#
*
*
0.6
0.4
0.2
RT Los
Ctrl
Ctrl
RT
Ctrl
RT
RT
RT Los
RT Los
15 wk
1 wk
3 wk
42-44
42-44
42-44
pERK2
GAPDH
36
36
36
*#
### Chart
| Category | |
|---|---|
| Control | 0.18436356309046992 |
| RT | 0.26409496110621655 |
| RT Los | 0.2837393083985032 |
| | None |
| Control | 0.10769545020239632 |
| RT | 0.12430960853023369 |
| RT Los | 0.18731376034901123 |
| | None |
| Control | 0.18666436797151015 |
| RT | 0.3061689898676477 |
| RT Los | 0.36992204623846303 |0.4
*
0.3
*
0.2
0.1
Ctrl
Ctrl
RT
RT
Ctrl
RT Los
RT Los
RT Los
RT
15 wk
1 wk
3 wk

## Slide 11
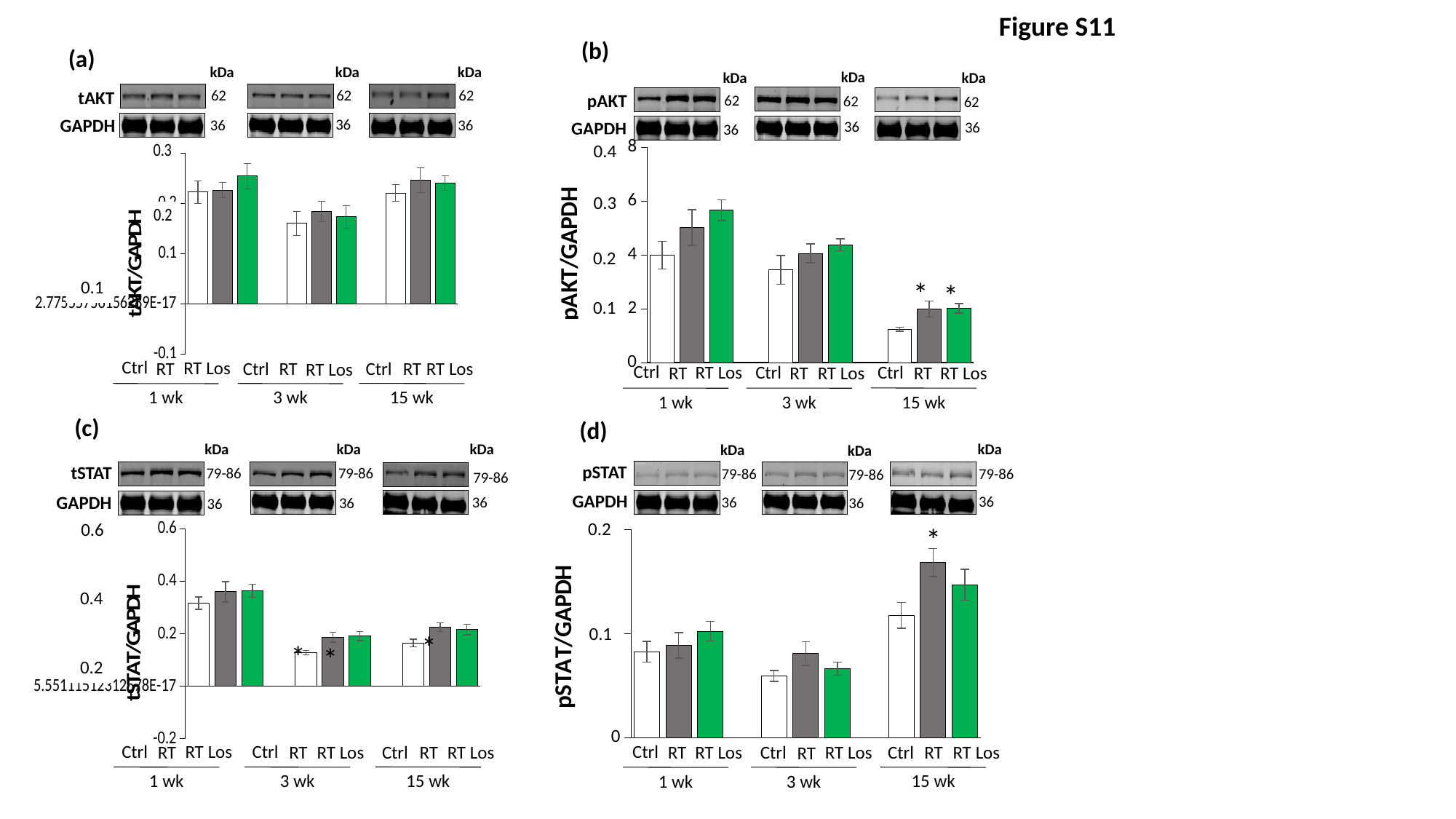

Figure S11
(b)
(a)
kDa
kDa
kDa
62
62
62
tAKT
GAPDH
36
36
36
### Chart
| Category | |
|---|---|
| Control | 0.22253284534898551 |
| RT | 0.22687926195136715 |
| RT Los | 0.2544695293377849 |
| | None |
| Control | 0.160516778487271 |
| RT | 0.1841085638904262 |
| RT Los | 0.17339181700400827 |
| | None |
| Control | 0.22103435641344824 |
| RT | 0.24668649916478702 |
| RT Los | 0.2411678493089581 |0.1
Ctrl
RT Los
Ctrl
RT Los
RT
Ctrl
RT
RT
RT Los
15 wk
1 wk
3 wk
kDa
kDa
kDa
pAKT
62
62
62
GAPDH
36
36
36
### Chart
| Category | |
|---|---|
| Control | 0.39996905382690756 |
| RT | 0.5027098391650242 |
| RT Los | 0.5674084136298858 |
| | None |
| Control | 0.345122522630116 |
| RT | 0.40660489661223825 |
| RT Los | 0.43848133876805134 |
| | None |
| Control | 0.12483978832054062 |
| RT | 0.1992133516030352 |
| RT Los | 0.2018961738088065 |*
*
0.4
0.3
0.2
0.1
Ctrl
Ctrl
RT Los
Ctrl
RT Los
RT Los
RT
RT
RT
15 wk
1 wk
3 wk
(c)
(d)
kDa
kDa
kDa
tSTAT
79-86
79-86
GAPDH
36
36
36
### Chart
| Category | |
|---|---|
| Control | 0.3174611937937354 |
| RT | 0.3605905620121522 |
| RT Los | 0.36432822116589714 |
| | None |
| Control | 0.12869862738474605 |
| RT | 0.18763814029715306 |
| RT Los | 0.19162815722647478 |
| | None |
| Control | 0.16571654047947026 |
| RT | 0.22621548943211833 |
| RT Los | 0.21661034182509922 |0.6
0.4
0.2
RT Los
Ctrl
Ctrl
RT Los
RT
Ctrl
RT
RT
RT Los
15 wk
1 wk
3 wk
kDa
kDa
kDa
pSTAT
79-86
79-86
79-86
GAPDH
36
36
36
### Chart
| Category | |
|---|---|
| Control | 0.08256487796746023 |
| RT | 0.08859590476042438 |
| RT Los | 0.1022097744149787 |
| | None |
| Control | 0.059341166834348154 |
| RT | 0.08085630084618821 |
| RT Los | 0.0663994245074703 |
| | None |
| Control | 0.11745651684603163 |
| RT | 0.16812010041451822 |
| RT Los | 0.1468748882706219 |0.2
0.1
Ctrl
RT Los
Ctrl
RT Los
RT
Ctrl
RT Los
RT
RT
15 wk
1 wk
3 wk
79-86
*
*
*
*

## Slide 12
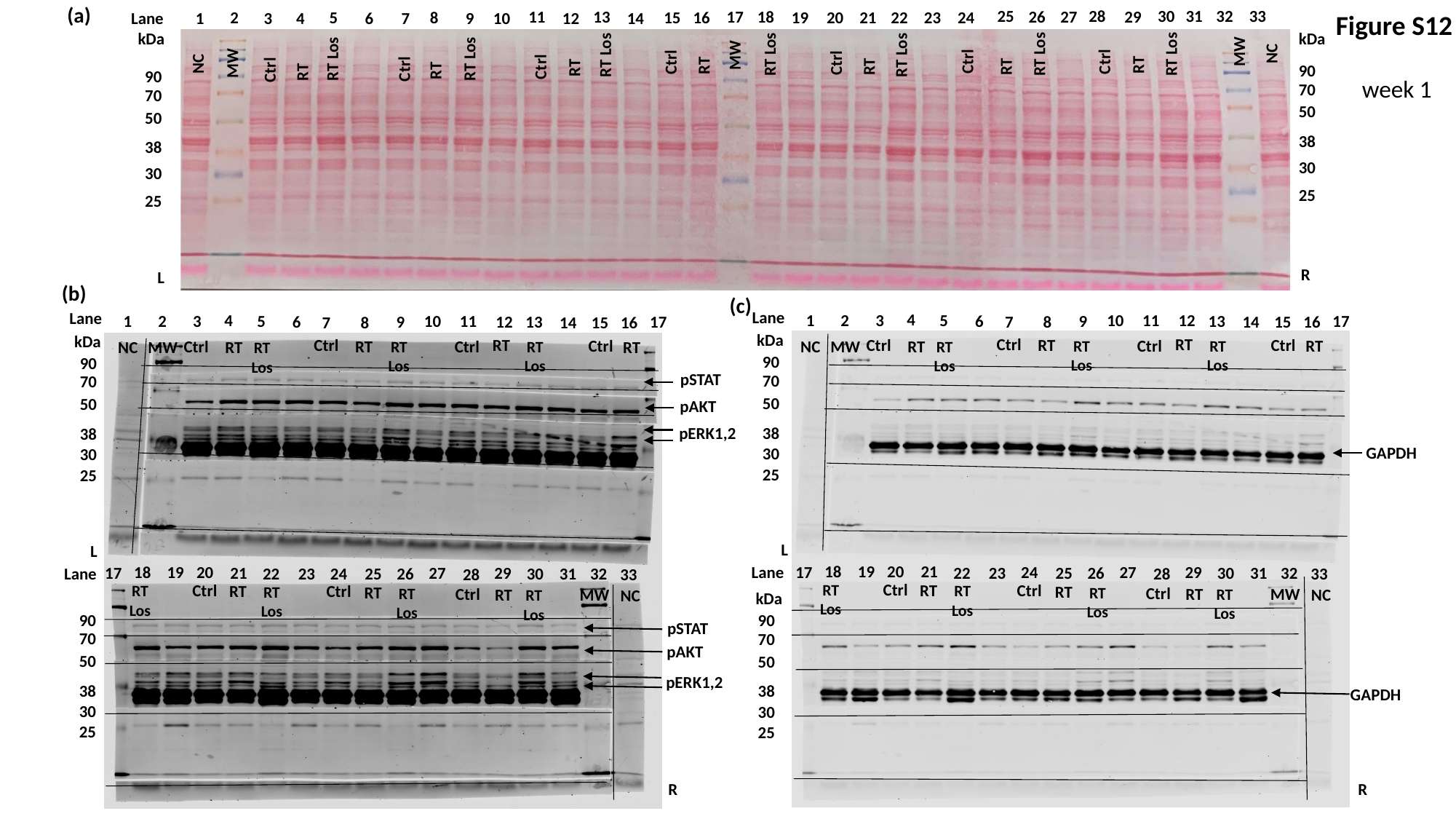

25
28
32
33
(a)
30
31
27
29
26
11
18
13
17
15
8
2
5
19
16
20
23
24
22
21
6
12
14
4
10
1
3
7
9
Lane
Figure S12
kDa
kDa
MW
NC
RT Los
RT Los
RT Los
MW
RT Los
RT Los
RT Los
RT Los
Ctrl
Ctrl
Ctrl
Ctrl
MW
NC
RT
RT
RT
RT
Ctrl
RT
Ctrl
Ctrl
90
RT
RT
90
week 1
70
70
50
50
38
38
30
30
25
25
R
L
(b)
(c)
Lane
Lane
4
1
11
3
2
10
5
12
4
1
11
3
6
17
13
9
2
10
5
14
12
7
15
16
8
6
17
13
9
14
7
15
16
8
kDa
kDa
RT
Ctrl
Ctrl
RT
Ctrl
Ctrl
RT
Ctrl
Ctrl
RT
Ctrl
RT
RT
MW
Ctrl
RT Los
RT Los
NC
RT
RT
MW
RT Los
RT Los
RT Los
NC
RT Los
90
90
pSTAT
70
70
50
50
pAKT
38
pERK1,2
38
GAPDH
30
30
25
25
L
L
19
20
18
21
17
27
29
24
Lane
19
20
18
25
31
30
32
21
22
23
26
17
27
29
Lane
28
24
25
31
30
32
33
22
23
26
28
33
Ctrl
RT
Ctrl
Ctrl
RT Los
RT
Ctrl
RT Los
RT Los
RT
RT Los
RT
Ctrl
RT Los
Ctrl
RT
MW
RT Los
RT
MW
RT Los
NC
RT Los
NC
kDa
90
90
pSTAT
70
70
pAKT
50
50
pERK1,2
38
38
GAPDH
30
30
25
25
R
R

## Slide 13
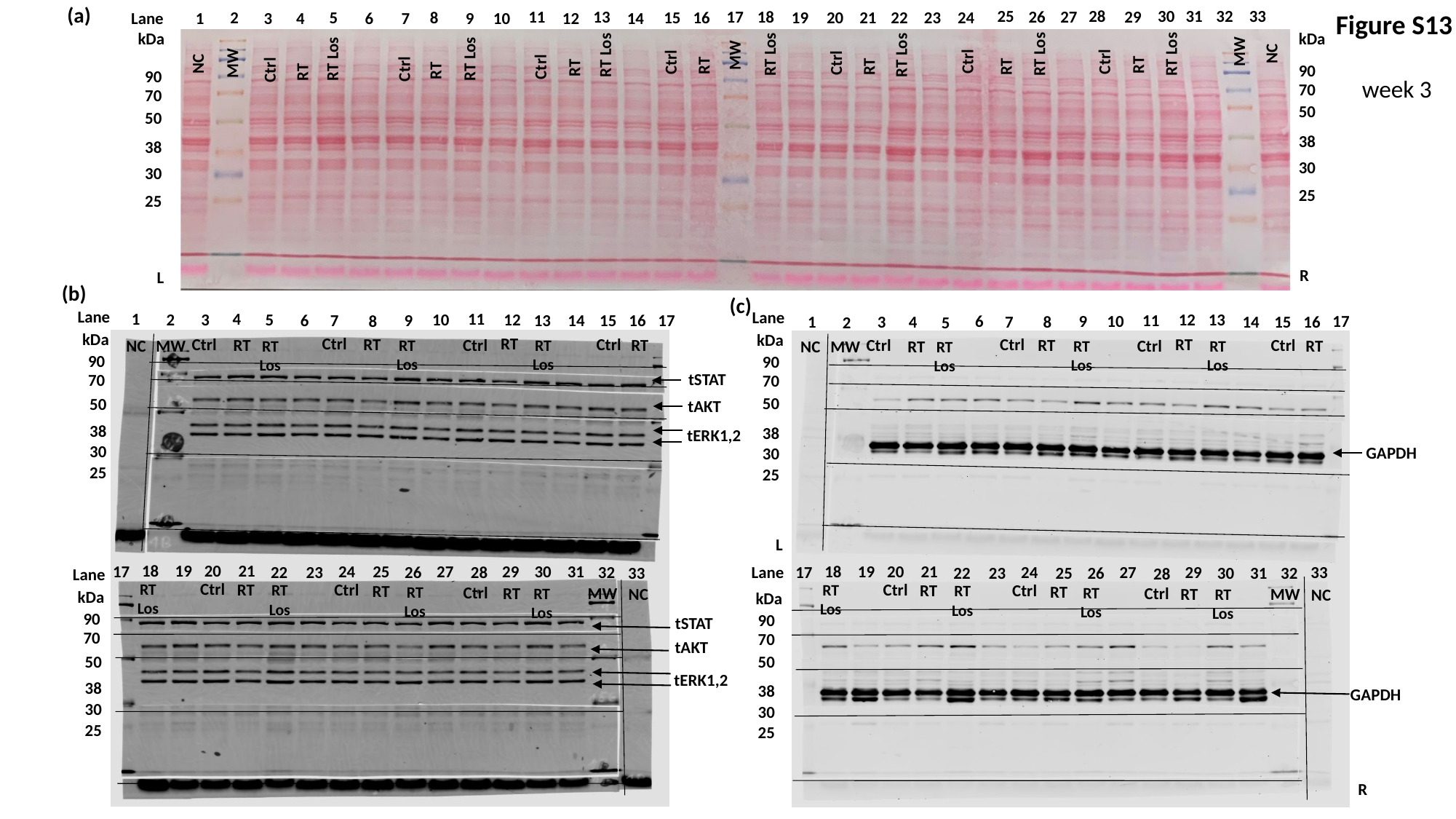

25
28
32
33
(a)
30
31
27
29
26
11
18
13
17
15
8
2
5
19
16
20
Figure S13
23
24
22
21
6
12
14
4
10
1
3
7
9
Lane
kDa
kDa
MW
NC
RT Los
RT Los
RT Los
MW
RT Los
RT Los
RT Los
RT Los
Ctrl
Ctrl
Ctrl
Ctrl
MW
NC
RT
RT
RT
RT
Ctrl
RT
Ctrl
Ctrl
90
RT
RT
90
week 3
70
70
50
50
38
38
30
30
25
25
R
L
(b)
(c)
Lane
Lane
4
1
11
3
12
2
10
5
12
13
6
17
13
9
11
14
7
15
16
8
10
6
17
9
14
7
15
16
4
8
1
3
2
5
kDa
kDa
RT
Ctrl
Ctrl
RT
Ctrl
RT
Ctrl
Ctrl
Ctrl
RT
RT
Ctrl
RT
MW
RT Los
RT Los
Ctrl
NC
RT
RT
MW
RT Los
RT Los
RT Los
NC
RT Los
90
90
tSTAT
70
70
50
50
tAKT
38
38
tERK1,2
30
GAPDH
30
25
25
L
19
20
18
21
17
27
29
19
20
18
24
25
31
21
30
32
33
22
23
26
17
27
29
24
Lane
28
25
31
30
32
33
22
23
26
28
Lane
Ctrl
Ctrl
RT
Ctrl
RT Los
RT
Ctrl
RT Los
RT Los
RT
RT Los
RT
Ctrl
RT Los
Ctrl
RT Los
RT
MW
RT
MW
RT Los
NC
RT Los
NC
kDa
kDa
90
90
tSTAT
70
70
tAKT
50
50
tERK1,2
38
38
GAPDH
30
30
25
25
R

## Slide 14
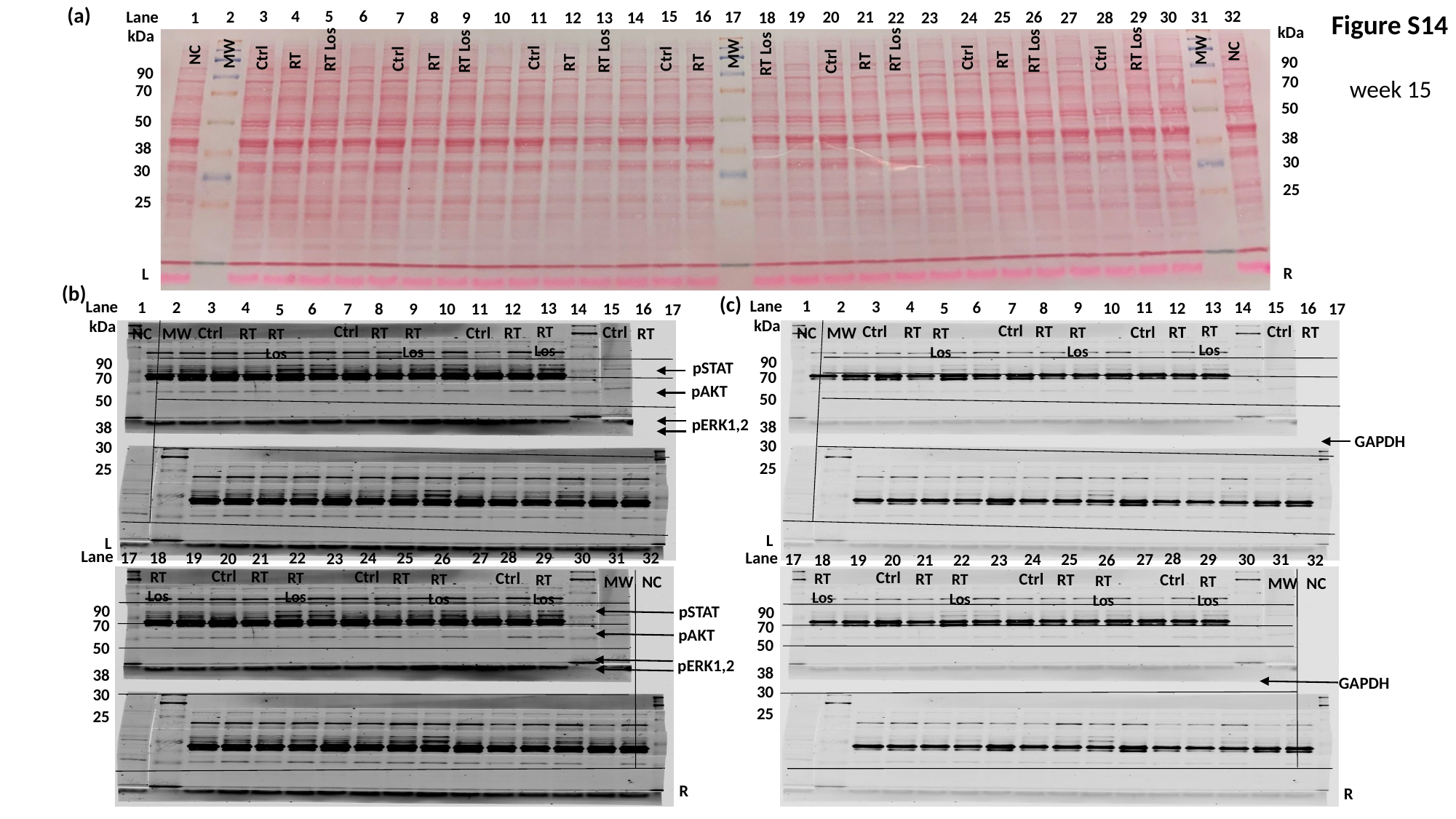

5
(a)
6
4
32
3
15
16
2
30
31
21
20
19
Lane
17
29
25
26
18
23
27
11
22
28
8
9
10
14
7
1
24
Figure S14
13
12
kDa
kDa
RT Los
MW
RT Los
RT Los
RT Los
RT Los
RT Los
NC
MW
MW
RT Los
NC
Ctrl
Ctrl
Ctrl
Ctrl
Ctrl
Ctrl
RT
RT
RT
Ctrl
90
RT
RT
RT
90
70
week 15
70
50
50
38
38
30
30
25
25
R
L
(b)
(c)
Lane
1
3
4
2
Lane
1
13
3
9
15
6
11
4
14
2
7
16
10
8
12
5
13
9
15
6
17
11
14
7
16
10
8
12
5
17
kDa
kDa
Ctrl
Ctrl
RT Los
Ctrl
RT
Ctrl
Ctrl
RT
Ctrl
RT
RT Los
RT
MW
Ctrl
RT
RT Los
NC
Ctrl
RT
RT
RT
RT Los
MW
RT Los
NC
RT Los
90
90
pSTAT
70
70
pAKT
50
50
pERK1,2
38
38
GAPDH
30
30
25
25
L
L
Lane
28
17
27
29
25
24
31
30
Lane
32
19
18
22
21
23
20
26
28
17
27
29
25
24
31
30
32
19
18
22
21
23
20
26
Ctrl
RT
Ctrl
RT Los
Ctrl
RT Los
RT
Ctrl
RT
Ctrl
RT Los
RT Los
RT
RT Los
Ctrl
RT Los
MW
RT Los
NC
RT Los
MW
NC
90
pSTAT
90
70
70
pAKT
50
50
pERK1,2
38
38
GAPDH
30
30
25
25
R
R

## Slide 15
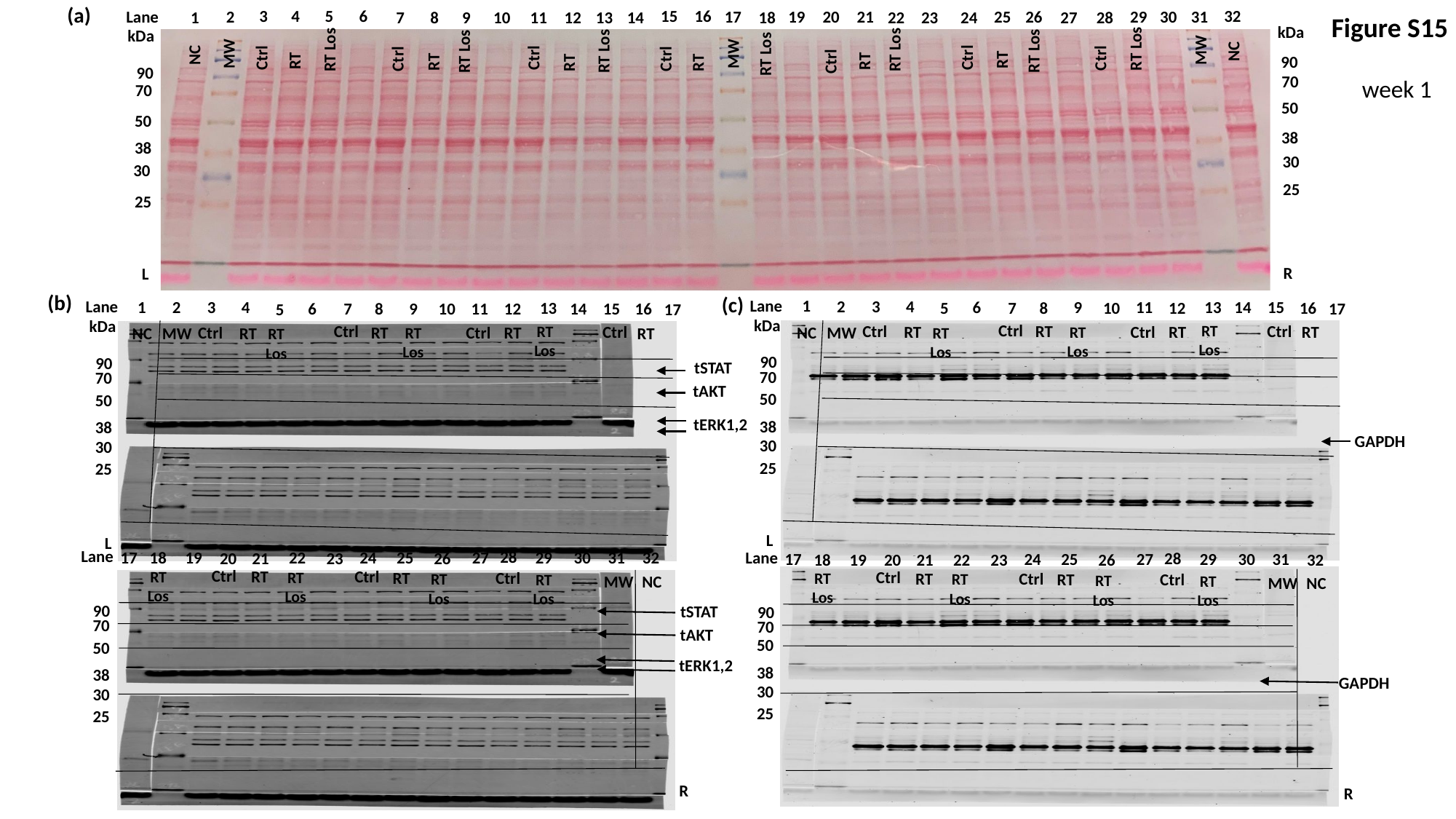

5
(a)
6
4
32
3
15
16
2
30
31
21
20
19
Lane
17
29
25
26
18
23
27
11
22
28
8
9
10
14
7
1
24
13
12
Figure S15
kDa
kDa
RT Los
MW
RT Los
RT Los
RT Los
RT Los
RT Los
NC
MW
MW
RT Los
NC
Ctrl
Ctrl
Ctrl
Ctrl
Ctrl
Ctrl
RT
RT
RT
Ctrl
90
RT
RT
RT
90
70
week 1
70
50
50
38
38
30
30
25
25
R
L
(b)
(c)
Lane
1
3
4
2
Lane
1
13
3
9
15
6
11
4
14
2
7
16
10
8
12
5
13
9
15
6
17
11
14
7
16
10
8
12
5
17
kDa
kDa
Ctrl
Ctrl
RT Los
Ctrl
RT
Ctrl
Ctrl
RT
Ctrl
RT
RT Los
RT
MW
Ctrl
RT
RT Los
NC
Ctrl
RT
RT
RT
RT Los
MW
RT Los
NC
RT Los
90
90
tSTAT
70
70
tAKT
50
50
tERK1,2
38
38
GAPDH
30
30
25
25
L
L
Lane
28
17
27
29
25
24
31
30
Lane
32
19
18
22
21
23
20
26
28
17
27
29
25
24
31
30
32
19
18
22
21
23
20
26
Ctrl
RT
Ctrl
RT Los
Ctrl
RT Los
RT
Ctrl
RT
Ctrl
RT Los
RT Los
RT
RT Los
Ctrl
RT Los
MW
RT Los
NC
RT Los
MW
NC
90
tSTAT
90
70
70
tAKT
50
50
tERK1,2
38
38
GAPDH
30
30
25
25
R
R

## Slide 16
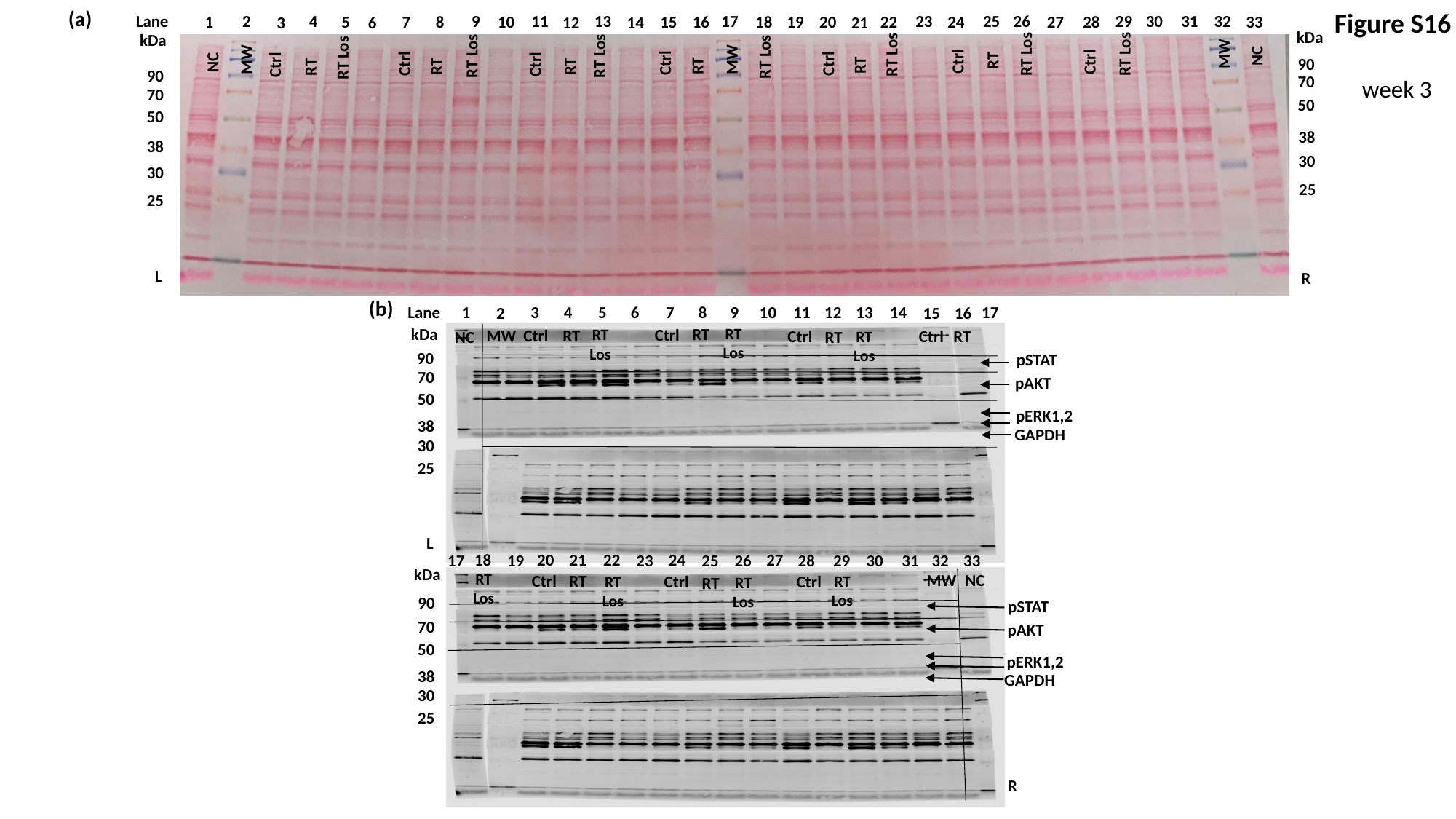

(a)
Figure S16
Lane
26
29
2
17
4
31
25
32
9
13
30
23
11
27
33
22
15
28
16
18
19
20
7
24
8
10
5
1
12
14
3
6
21
kDa
kDa
MW
RT Los
RT Los
RT Los
NC
RT Los
RT Los
RT Los
RT Los
MW
MW
RT
Ctrl
Ctrl
NC
Ctrl
Ctrl
Ctrl
90
Ctrl
Ctrl
RT
RT
RT
RT
RT
90
70
week 3
70
50
50
38
38
30
30
25
25
L
R
(b)
4
7
9
10
11
Lane
5
1
8
12
3
14
17
6
13
2
15
16
kDa
RT
RT Los
Ctrl
RT
RT Los
MW
Ctrl
Ctrl
RT
Ctrl
RT Los
RT
NC
90
pSTAT
70
pAKT
50
pERK1,2
38
GAPDH
30
25
L
21
22
27
18
20
24
28
17
26
30
31
32
25
33
23
19
29
kDa
RT Los
MW
NC
Ctrl
RT
Ctrl
Ctrl
RT Los
RT Los
RT
RT Los
90
pSTAT
70
pAKT
50
pERK1,2
38
GAPDH
30
25
R

## Slide 17
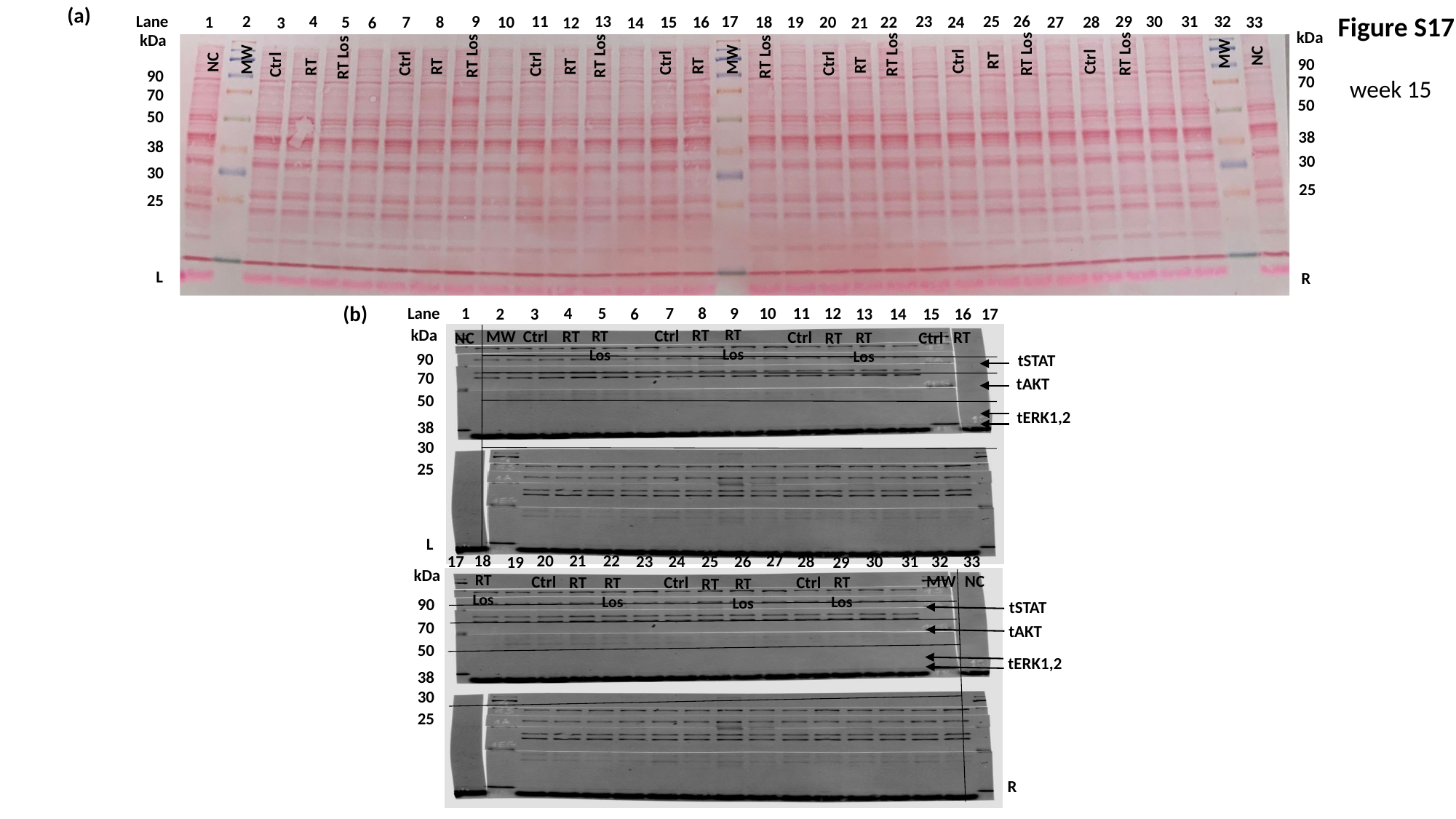

(a)
Figure S17
Lane
26
29
2
17
4
31
25
32
9
13
30
23
11
27
33
22
15
28
16
18
19
20
7
24
8
10
5
1
12
14
3
6
21
kDa
kDa
MW
RT Los
RT Los
RT Los
NC
RT Los
RT Los
RT Los
RT Los
MW
MW
RT
Ctrl
Ctrl
NC
Ctrl
Ctrl
Ctrl
90
Ctrl
Ctrl
RT
RT
RT
RT
RT
90
70
week 15
70
50
50
38
38
30
30
25
25
L
R
(b)
4
7
9
10
11
Lane
5
1
8
12
3
14
17
6
13
2
15
16
kDa
RT
RT Los
Ctrl
RT
RT Los
MW
Ctrl
Ctrl
RT
Ctrl
RT Los
RT
NC
90
tSTAT
70
tAKT
50
tERK1,2
38
30
25
L
21
22
27
18
20
24
28
17
26
30
31
32
25
33
23
19
29
kDa
RT Los
MW
NC
Ctrl
RT
Ctrl
Ctrl
RT Los
RT Los
RT
RT Los
90
tSTAT
70
tAKT
50
tERK1,2
38
30
25
R
